# The mouse retinal pigment epithelium mounts an innate immune defense response following retinal detachment

**DOI:** 10.1186/s12974-024-03062-2

**Published:** 2024-03-25

**Authors:** Steven F. Abcouwer, Bruna Miglioranza Scavuzzi, Phillip E. Kish, Dejuan Kong, Sumathi Shanmugam, Xuan An Le, Jingyu Yao, Heather Hager, David N Zacks

**Affiliations:** Department of Ophthalmology and Visual Sciences, Kellogg Eye Center, University of Michigan Medicine, 1000 Wall Street, Ann Arbor, MI 48105 USA

## Abstract

The retinal pigment epithelium (RPE) maintains photoreceptor viability and function, completes the visual cycle, and forms the outer blood-retinal barrier (oBRB). Loss of RPE function gives rise to several monogenic retinal dystrophies and contributes to age-related macular degeneration. Retinal detachment (RD) causes separation of the neurosensory retina from the underlying RPE, disrupting the functional and metabolic relationships between these layers. Although the retinal response to RD is highly studied, little is known about how the RPE responds to loss of this interaction. RNA sequencing (RNA-Seq) was used to compare normal and detached RPE in the C57BL6/J mouse. The naïve mouse RPE transcriptome was compared to previously published RPE signature gene lists and from the union of these 14 genes (*Bmp4*, *Crim1*, *Degs1*, *Gja1*, *Itgav*, *Mfap3l*, *Pdpn*, *Ptgds*, *Rbp1*, *Rnf13*, *Rpe65*, *Slc4a2*, *Sulf1 and Ttr*) representing a core signature gene set applicable across rodent and human RPE was derived. Gene ontology enrichment analysis (GOEA) of the mouse RPE transcriptome identified expected RPE features and functions, such as pigmentation, phagocytosis, lysosomal and proteasomal degradation of proteins, and barrier function. Differentially expressed genes (DEG) at 1 and 7 days post retinal detachment (dprd) were defined as mRNA with a significant (p_adj_≤0.05) fold change (FC) of 0.67 ≥ FC ≥ 1.5 in detached versus naïve RPE. The RPE transcriptome exhibited dramatic changes at 1 dprd, with 2297 DEG identified. The KEGG pathways and biological process GO groups related to innate immune responses were significantly enriched. Lipocalin 2 (*Lcn2*) and several chemokines were upregulated, while numerous genes related to RPE functions, such as pigment synthesis, visual cycle, phagocytosis, and tight junctions were downregulated at 1 dprd. The response was largely transient, with only 18 significant DEG identified at 7 dprd, including upregulation of complement gene *C4b*. Validation studies confirmed RNA-Seq results. Thus, the RPE quickly downregulates cell-specific functions and mounts an innate immune defense response following RD. Our data demonstrate that the RPE contributes to the inflammatory response to RD and may play a role in attraction of immune cells to the subretinal space.

**Supplementary Information:**

The online version contains supplementary material available at 10.1186/s12974-024-03062-2.

## Introduction

The retinal pigment epithelium (RPE) consists of a monolayer of polarized epithelial cells located between the neural retina and the choroid vasculature. RPE cells are specialized phagocytes that engulf and digest shed tips of photoreceptor (PR) outer segments (OS) and recycle 11-cis-retinal and metabolites back to the PRs, thus supporting the continual production of PR inner segments and the visual phototransduction cycle [1, 2]. The RPE also constitutes the outer blood-retinal barrier (oBRB), by contributing to the formation and maintenance of the Bruch’s membrane at its base and by forming intercellular tight junctions [3, 4], which together control the movement of ions, metabolites, and cells between the choroidal vasculature and the subretinal space [3]. The RPE is also instrumental in both formation and maintenance of the choriocapillaris, which is dependent on continual secretion of vascular endothelial growth factor A by the RPE [5]. The RPE also plays a major role in maintaining ocular immune privilege, both by providing a barrier to immune cell transit and by producing a number of immune-suppressing molecules [6].

Retinal detachment (RD) is a serious ocular condition characterized by the separation of the neurosensory retina from the underlying RPE and choroid, disrupting the functional and morphologic relationship between these layers [7]. Rhegmatogenous RD (RRD), the most common type of RD, occurs when a full-thickness break forms in the neurosensory retina leading to the influx of fluid from the vitreous cavity and separation of the retina and RPE. In addition, tractional and exudative detachments also cause physical retina-RPE separations, with subretinal accumulation of vitreous humor. Thus, RD disrupts the close association between RPE and PR. Experimental in vivo models, such as mouse models, are widely used to study the mechanisms underlying RD and potential therapeutic interventions [8–10]. In these models, solution containing high molecular weight polymeric hyaluronic acid (HA), saline, or balanced salt solutions are surgically injected between the neural retina and RPE to create a separation that mimics clinical RD. Hyaluronic acid is commonly used in RD models because of its ability to prevent spontaneous retinal re-attachment, lack of toxicity, and natural presence in the vitreous humor [10].

Using such models, we and others have described a rapid innate immune response to RD, with microglial activation and the attraction of both microglia and systemic immune cells to the subretinal space caused by RD [11, 12]. However, the role of the RPE in this inflammatory response has not been well considered. In age-related macular degeneration (AMD) pathology, a sight-threatening disease involving RPE degeneration and subretinal inflammation, a conventional view is that dysfunction of the RPE causes accumulation of OS debris in the subretinal space, thus leading to attraction and inflammatory activation of both resident and systemic innate immune cells [13]. However, increasingly it is recognized that the RPE itself can initiate an innate immune response. The RPE responds to inflammatory cytokines and is well equipped with pattern recognition receptors, including toll-like receptors, that can detect both intracellular and extracellular signals that stimulate inflammasome activation and cytokine expression [14, 15]. Such intrinsic innate immune responses by the RPE are now hypothesized to be a key initiator of RPE pathology [16]. In addition, it has recently been observed that a lack of anti-inflammatory signaling through TAM receptors, Mer tyrosine kinase (Mertk) and Tyro3 protein kinase, can also lead to an innate immune response of RPE cells [17]. Thus, it is feasible that an intrinsic RPE innate immune response causes RPE dysfunction and initiates the innate immune cell response following RD. Using in situ hybridization Rattner and co-workers [18] demonstrated that the RPE exhibited downregulation of RPE-specific genes and increased expression of innate immune-related genes following both light damage and RD. These authors provided evidence that the RPE response following light damage was dependent upon release of a paracrine factor released by the retina. However, this finding has not been confirmed or expanded upon.

In this study, we used a mouse model of RD, a previously published method of simultaneous RPE cell isolation and RNA stabilization (SRIRS) [19] and bulk RNA sequencing (RNA-Seq) to examine the transcriptomic response of the RPE at 1 and 7 days post RD (dprd). Because the effects of RD on the contralateral eye have not been defined, RD groups were compared to matched naïve (Nv) RPE samples, which allowed us to also define a normal mouse RPE transcriptome. We compared this naïve mouse RPE transcriptome to other studies defining RPE signature genes and highlighted genes of importance to key RPE functions. Enrichment analyses identified expected RPE features and functions, such as pigmentation, phagocytosis, lysosomal and proteasomal degradation of proteins, and barrier function, as well as enrichment of genes involved in mitochondrial respiration, protein synthesis and protein processing in the endoplasmic reticulum. Differential gene expression comparisons between Nv and RD RPE revealed a rapid downregulation of many RPE marker genes and upregulation of many genes involved in innate immune defense responses. The RPE response to RD declined with time, as the vast majority of DEG at 1 dprd were altered at 7 dprd but were no longer significantly different from the Nv group. The small number of significant (p_adj_≤0.05) DEG identified at 7 dprd include C4b, encoding complement factor C4, which was detected in the subretinal space (SRS) associated with subretinal immune cells. The results suggest that the RPE plays a heretofore unappreciated role in the innate immune response to RD.

## Methods

### Mouse RD model

All animal experiments were conducted in compliance with the guidelines set forth by the Institutional Animal Care & Use Committee (IACUC) of the University of Michigan and adhered to the Association for Research in Vision and Ophthalmology (ARVO) Statement for the Use of Animals in Ophthalmic and Visual Research.

Male C57BL6/J mice, aged 8 to 10 weeks, were obtained from The Jackson Laboratory. Lipocalin-2 (Lcn2) knockout (KO) mouse [20] on a C57BL/6J background (Jackson Laboratories strain #024530) was maintained as homozygous. Both male and female Lcn2 KO mice were utilized to validate anti-LCN2 antibody results. All mice were housed in a temperature-controlled environment (25 °C) with a 12-hour light-dark cycle (6:00 am to 6:00 pm). Retinal detachments were created as previously described [12]. All detachments were performed in the morning (between 9:00 am and 11:00 am) and under anesthesia, using a mixture of ketamine (80 mg/kg) and xylazine (10 mg/kg). Pupils were dilated with an ophthalmic drop containing a combination of phenylephrine 2.5% and tropicamide 1%. A sclerotomy was created approximately 1 mm posterior to the limbus using a 30-gauge needle. Subsequently, 1% sodium hyaluronate (Healon®PRO; catalog # 10,240,011, Johnson & Johnson Vision) was carefully injected into the subretinal space until approximately 50% of the retina became detached from the RPE. The eyes were enucleated 24 h (1 dprd) or 168 h (7 dprd) after RD.

### Sample harvesting

Anterior segment and lenses were removed from enucleated eyes. Retinas were removed from eyecups, immediately placed in liquid nitrogen, and stored at -80 °C until further processing. RPE RNA was isolated from eyecups based on a previously detailed protocol [21]. In short, after removing the retina the remaining posterior eyecup was quickly washed in PBS buffer and then immediately transferred into a 1.5 mL microcentrifuge tube containing 300 μL of RNAprotect^®^ Cell Reagent (catalog# 76,526, Qiagen Sciences). The eyecup was incubated for 20 min at room temperature to allow dissolution of the RPE, briefly vortexed, and the eyecup was removed. Centrifugation was then performed for 5 min at 900 x g to pellet RNA-containing reverse micelles. The pellets were darkly pigmented due to precipitation of melanin crystals (melanosomes). RPE-enriched RNA pellets were stored at -80 °C until subjected to total RNA purification.

### RNA purification

Retinal tissue (cleaned of vitreous and RPE) and SRIRS RPE RNA-containing pellets were dissolved in RLT Buffer (Qiagen), and total RNA was obtained using the column-based RNA purification kit, RNeasy Plus Micro (Qiagen). RNA from choroid/sclera tissue (empty eyecups following SRIRS RPE RNA extraction) was extracted using TRIzol reagent (Invitrogen) following supplier’s instructions. RNA concentration and quality were assessed using the RNA ScreenTape system (Agilent Technologies) and Quant-iT™ RiboGreen® RNA quantitation kit (Invitrogen).

### RNA sequencing

TruSeq stranded mRNA kit (Illumina) was used for cDNA library generation according to supplier’s instructions, with 1000 ng of total RNA input per sample. cDNA preparation was conducted by MedGenome utilizing the TruSeq mRNA stranded kit (Illumina). Initially, poly-A containing mRNA molecules were purified using poly-dT oligo attached magnetic beads. Subsequently, cDNA was synthesized according to a method described in Illumina TruSeq Stranded mRNA Reference Guide (https://support.illumina.com/downloads/truseq-stranded-mrna-reference-guide-1000000040498.html). One hundred bp single-end reads were generated using NovaSeq sequencer (Illumina). Contaminating sequences, such as ribosomal (rRNA), transfer (tRNA), mitochondrial (mtDNA) and adapter sequences, were removed using Bowtie2 (v2.5.1). The remaining read sequences were aligned to the reference mouse genome obtained from the Ensembl database (genome-build GRCm38.p6; genome-version GRCm38; genome-build-accession NCBI: GCA_000001635.8) using the STAR (v2.7.3a) aligner. HTSeq (v0.11.2) was used to estimate raw read counts, which were then normalized using DESeq2. Cufflinks (v2.2.1) was employed to estimate gene expression, and the expression values were reported as fragments per kilobase of transcript per million mapped reads (FPKM) and transcript per million reads (TPM) units for each gene. DESeq2, an R Bioconductor package, was utilized for conducting differential expression analysis. The comparison was made between the RD groups (RD1 and RD7) and the Nv group to identify the differential expression patterns. To determine differentially expressed genes (DEG), genes with an adjusted *p*-value (Benjamini and Hochberg method) less than 0.05 were considered significant and those meeting cutoffs of a fold change (FC) greater than or equal to 1.5 (upregulated), or less than or equal to 0.67 (downregulated) were retained. Gene Ontology (GO), KEGG, K-means clustering, and enrichment analysis were performed using the iDEP and ShinyGO-0.77 web applications [22–24]. Heatmaps were prepared using the Flaski heatmap application [25] with the Ward’s hierarchical clustering method [26].

One naïve RPE sample (Nv1) in the original naïve group (*n* = 6) was excluded from the analysis. This sample did not exhibit a 3 standard deviation separation from its group in PCA analysis (as recommended at https://www.biostars.org/p/281767/). However, we applied Grubbs outlier analyses (https://www.graphpad.com/quickcalcs/grubbs1/) to the 250 genes exhibiting the highest variances in TPMs in the Nv group and identified Nv1 TPM values as being consistent outliers (alpha = 0.05). These 250 genes exhibited relatively high Nv1 TPM values for genes that are highly expressed in PR and other retinal cell types, suggesting that Nv1 sample was contaminated with a relatively high amount of retinal tissue. Therefore, it was omitted from the DEG analysis.

### qRT-PCR

RNA sequencing results were validated by qRT-PCR analysis of selected genes in an independent set of samples. cDNA was synthesized from total RNA using random hexamers and oligo-dT primers (Omniscript RT kit, Qiagen). Duplex qRT-PCR was performed using TaqMan™ Universal PCR Master Mix (Thermo Fisher Scientific) with gene-specific FAM-labeled TaqMan assays (Thermo Fisher Scientific), including: *A2m* (Assay ID: Mm00558642_m1), *C1qa* (Mm00432142_m1), *C4b* (Mm00437893_g1), *Ccl8* (Assay ID: Mm01297183_m1), *Grem1* (Mm00488615_s1), Gsto1 (Mm00599566_m1); *Lcn2* (Mm01324470_m1), *Lrat* (Mm00469972_m1), *Mpeg1* (Mm01222137_g1), *Myrip* (Mm00460563_m1) and (Mm00460566_m1), *Rpe65* (Mm00504133_m1), *S100a8* (Mm00496696_g1), *Serpine3* (Mm01310498_m1), *Snhg11* (Mm04212327_m1) and (Mm00558874_m1), *Timp1* (Mm01341361_m1), *Tsc22d3* (Mm00726417_s1), *Wfikkn2* (Mm00725281_m1), *Zbp1* (Mm01247052_m1). Gene transcript abundance was normalized to that of an endogenous housekeeping gene, by duplex assays using VIC-labeled TaqMan assay for *Actg1* (Mm01963702_s1) or *18 S* (Hs99999901_s1). Reactions were performed and fluorescence was monitored using a Real-Time PCR System (CFX384 Touch, BioRad). Relative normalized mRNA levels were calculated using the ΔΔC_t_ method.

QRT-PCR was also used to evaluate contamination with non-RPE RNA in the RPE-SRIRS preparations, by examining enrichment of an RPE-specific mRNA (*Rpe65* Mm00504133_m1), and contamination with endothelial (*Pecam1*, Mm01242576_m1), choroid endothelial (*Cdh5*, Mm00486938_m1), choroid/sclera (*Col1a1*, Mm00801666_g1) and retinal cell (*Rlbp1*, Mm00445129_m1; *Rho*, Mm00520345_m1) RNA contamination as per [27]. 18 S rRNA (Hs99999901_s1) was used as the internal control in duplex analysis.

### Western blot analysis

Protein was extracted from the RPE/choroid, according to a previously described methodology [28] and protein concentrations were measured by Pierce™ BCA protein assay kit (Thermo Fisher Scientific). Subsequently, 10 μg of protein was loaded into each well, separated on a 4–12% Bis-Tris SDS-PAGE gel (NuPAGE, Invitrogen), and transferred to a polyvinylidene fluoride membrane (Bio-Rad). After blocking with 5% bovine serum albumin (BSA, Sigma) in Tris-buffered saline (TBS, Bio-Rad), membranes were incubated overnight with primary antibodies: anti-mouse LCN2/NGAL (1:1000; catalog# AF1857, R&D Systems), or anti-α-Tubulin (1:1000; catalog# T6199, Millipore Sigma), used as a loading control. After three washes with 0.1% Tween-20 in TBS, incubated for 1 h with appropriate secondary antibody, either horseradish peroxidase (HRP)-conjugated anti-goat IgG (1:7500; Jackson ImmunoResearch), or HRP-conjugated anti-mouse IgG (1:8000; GE healthcare Lifesciences). Membranes were developed using SuperSignal^™^ West Pico PLUS chemiluminescent substrate (Thermo Fisher Scientific). Images were acquired using an Azure c500 or Azure 600 gel imager with cSeries Capture Software (Azure Biosystems).

### Intraocular fluid collection and western blotting

Intraocular fluid was collected as previously described [29] with small modifications for the evaluation of secreted proteins C4 and SERPINE3. Briefly, 4 eyeballs were enucleated and immersed in PBS, and excessive surrounding tissues were removed. Eyeballs were transferred into 350 μL of PBS, containing protease inhibitors, and cornea, optic nerve and lens were removed under the microscope to create an eyecup. The eyecup samples, including choroid and sclera, were minced, and centrifuged at 300 x g for 10 min at 4 °C and supernatant containing aqueous, vitreous, and subretinal fluid was collected. The tissue pellet was stored, and the supernatant was transferred into another 1.5 mL tube, which was centrifuged once more at 10,000 x g for 30 min at 4 °C. The supernatant was collected for further processing. For C4 evaluation, protein samples were separated in non-reducing conditions and separated on a non-reducing Bis-Tris buffered 4–12% PAGE gel (NuPAGE, Thermo Fisher Scientific) prior to immunoblotting. Primary antibodies employed were anti-C4 mAb (rat clone 16D2, 1:1000, Hycult Biotech), anti-SERPINE3 rabbit pAb (1:1000, Atlas Antibodies) and anti-SERPINE3 rabbit pAb (1:1000, Boster Biological Technology). Secondary antibodies employed were either HRP-conjugated anti-rabbit IgG (1:2000, Cell Signaling Technology), or HRP-conjugated anti-rat IgG (1:2000, Thermo Fisher Scientific).

### Immunofluorescence analysis of retinal sections

Immunofluorescence (IF) analysis was performed on retinal cryosections obtained from naïve C57BL/6J mouse, human, Brown Norway rat, pig, and rhesus monkey eyes. For the preparation of mouse and rat retinal sections, eyes were enucleated and subsequently fixed in a 4% paraformaldehyde (PFA) solution in PBS at room temperature (RT) for 1 h and 30 min. Pig eyes (obtained from a local abattoir) were enucleated and fixed in 4% PFA-PBS solution, overnight at 4 °C. For embedding of eyes, following the removal of the anterior segments, the posterior eye cups underwent a series of cryoprotection steps in PBS solutions with increasing sucrose concentrations, were embedded in a mixture consisting of a 1:1 ratio of 20% sucrose and optimal cutting temperature compound (OTC, Tissue-Tek 4583; Sakura Finetek) and sectioned at 10 μm thickness. Frozen eye sections from rhesus monkeys and postmortem humans were gifts from Dr. Bret A. Hughes (University of Michigan Medicine Kellogg Eye Center) and underwent processing similar to the pig eyes. Eye sections were subjected to blocking in TBS with 10% Donkey serum and 0.3% Triton X-100 (TBST) at RT for one hour, and incubated overnight at 4 °C with a mixture of primary antibodies in blocking buffer, including: Anti-GSTO1 rabbit pAb (dilution 1:100, Proteintech), mouse anti-RPE65 mAb (clone 8B11.37, dilution 1:250, a gift from Dr. Debra Thompson, University of Michigan Medicine Kellogg Eye Center), goat anti-mouse LCN2/NGAL pAb (1:1000; catalog# AF1857, R&D Systems), goat anti-serum raised against human C4 (1:25, Complement Technology, Inc.), rat anti-C4 mAb (clone 16D2, 1:1000, Hycult Biotech) and rabbit anti-AIF-1/Iba1 pAb (1:100, Novus Biologicals, NB100-1028). Following four 10 min washes in TBST, the sections were incubated with donkey anti-rabbit IgG AF488 (dilution 1:1000, Invitrogen) and donkey anti-mouse IgG AF594 or AFplus488 (dilution 1:1000, Invitrogen) secondary antibodies, plus Hoechst nucleic acid stain (dilution 1:1000, Invitrogen) and mounted using Prolong Gold Antifade (Invitrogen). Confocal microscopic imaging was performed using a STELLARIS 8 FALCON (Leica Microsystems Inc.) with a 40x objective lens and employing a consistent detection gain setting for each comparative section. To ensure specificity of the immunostaining, all experimental samples included negative controls, where the primary antibody was substituted with non-immune host IgG (1 mg/mL).

### Statistical analysis

Unless otherwise specified, data are presented as the mean value ± standard error of the mean (SEM). All statistical analyses, other than RNA-Seq DEG analysis, were performed using Prism 10.0 statistical software (GraphPad). One-way ANOVA with Tukey’s or Dunnett’s multiple comparisons test was used to evaluate statistical differences between groups.

## Results

### Defining the mouse RPE transcriptome

We and others previously described numerous gene expression changes in the retina following detachment from the RPE [30–32]. However, the overall transcriptional response of the RPE after retinal detachment has not been examined. We hypothesized that retinal detachment would lead to considerable gene expression changes in RPE that has been separated from the neural retina and is no longer in close association with PR outer segments. Previous studies showed that the simultaneous RPE isolation and RNA stabilization (SRIRS) method of Xin-Zhao and co-workers [19] provided highly enriched RPE RNA from the mouse eyecup. This method has the advantage of obtaining highly enriched RPE RNA and minimizing the chances for transcriptomic changes during isolation. Preliminary studies indicated that in our hands the SRIRS method provided RNA preparations greatly enriched in mouse RPE RNA (Supplemental Figure S1). This was demonstrated by qRT-PCR analysis showing enrichment of the RPE marker gene *Rpe65* (47-fold RPE > retina, *p* < 0.0001), as well as de-enrichment of rod PR-specific mRNA *Rho* (0.1317-fold, RPE < retina, *p* < 0.0001) the endothelial cell-specific mRNAs cadherin 5 (*Cdh5*) mRNA (0.034-fold, RPE < retina, *p* = 0.015) and platelet and endothelial cell adhesion molecule 1 (*Pecam1*, 0.045-fold, RPE < retina, *p* < 0.0001) mRNA (Supplemental Figures S1B-F). *Rhd5* and *Pecam1* are expressed in endothelial cells in both retinal vasculature and the choriocapillaris [33, 34]. Because expression of Collagen Type I Alpha 1 Chain is particularly high in choroidal stromal cells [33], the levels of *Col1a1* mRNA were also compared in the RPE SRIRS samples and RNA isolated from choroid/sclera tissue. This showed 187-fold higher level of *Col1a1* mRNA in choroid/sclera than in the RPE SRIRS preparation (RPE < choroid/sclera, *p* < 0.0001) (Supplemental Figure S1G), further ruling out any appreciable contamination from choroid RNA sources. We therefore used this method to isolate RPE RNA from age and sex (male) matched naïve C57BL/6J mouse eyecups and from eyes with partially detached retinas at 1 dprd and 7 dprd, all harvested at the same time of day (from 9:00 am to 11:00 am). Naïve eyes were chosen as the control group because the effects of RD on gene expression in the contralateral (fellow) eye have not been defined. Instead, we compared the RD groups to matched naïve (Nv) RPE samples, which allowed us to also define a normal mouse RPE transcriptome. Corresponding retinas were obtained from these same eyes and subjected to RNA-Seq analysis (reported elsewhere).

To define mouse RPE transcriptome based upon highly expressed mRNAs, mean transcripts per million (TPM) of mapped sequence reads for the Nv group were used to derive a list of 2671 genes with mean TPM ≥ 45, which represented the top 1/8th of 21,558 total detected genes in the Nv samples (Supplemental Table S1A). This emulates the strategy employed by Bergen’s group, who defined RPE signature genes as the top 10% of highly expressed transcripts [35]. The slightly less restrictive top 12.5% cutoff value was chosen because several previously published RPE signature genes were excluded when a top 10% cutoff was applied.

**Fig. 1:**
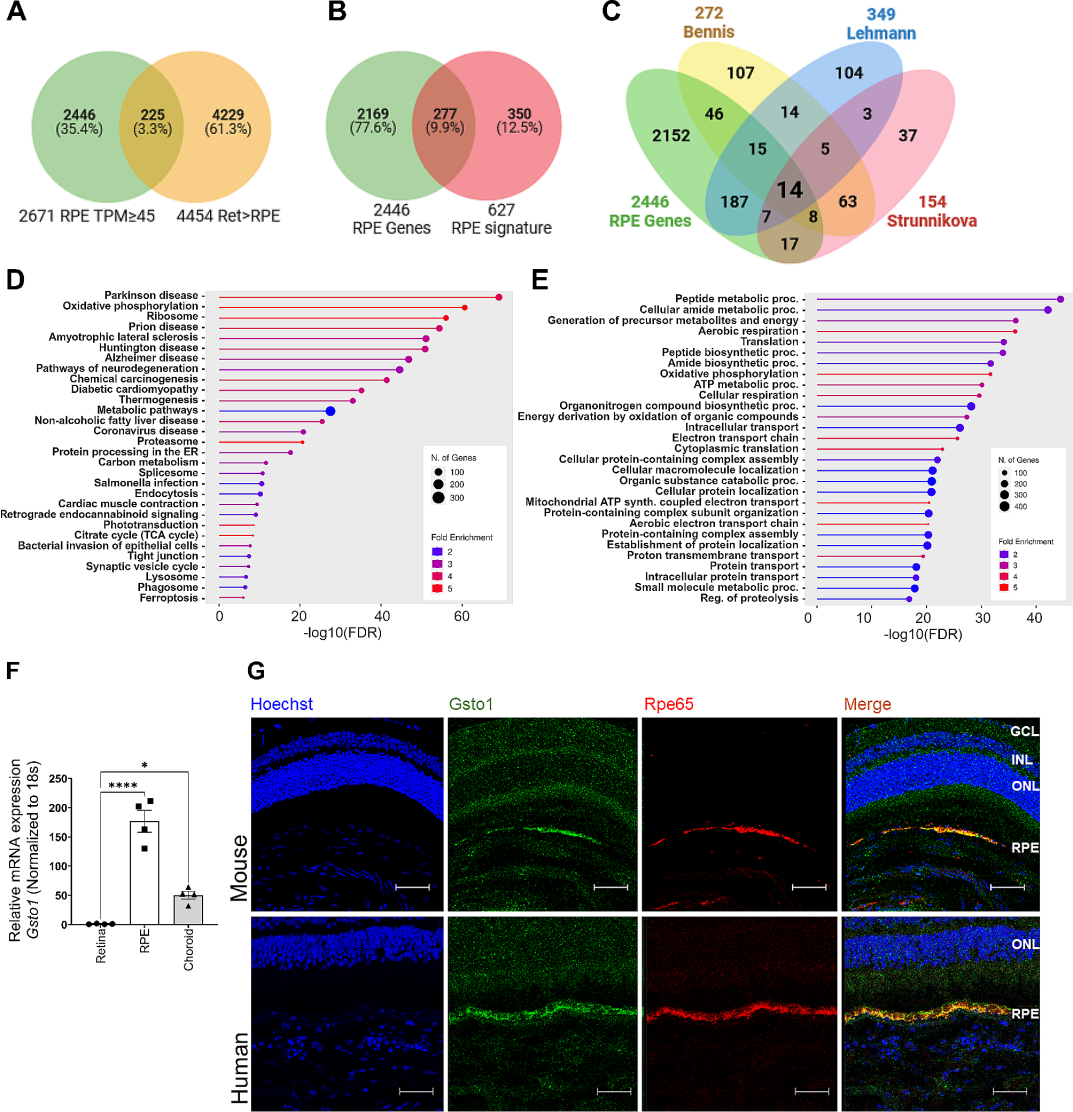
Analysis of the mouse RPE transcriptome. (**A**) Venn diagram showing comparison of RPE DEGs with TPM greater than 45 (total = 2671) and retina DEG with 2-fold greater transcript abundance in retina than the RPE padj ≤ 0.05 (total = 4454), and an overlap of 225 genes. (**B**) Venn diagram showing comparison of the 2446 gene mouse RPE transcriptome identified in the present study and the 627 RPE signature genes identified in three previous publications [33, 35, 36], and an intersection of 277 genes. (**C**) Venn diagram showing comparison of the RPE transcriptome identified in the present study and the lists identified in the works of Bennis, Lehmann and Strunnikova [33, 35, 36], with 14 genes common to the mouse RPE transcriptome and all three RPE signature gene lists. (**D**) Kyoto Encyclopedia of Genes and Genomes (KEGG) pathway enrichment analysis of 2446 gene naïve RPE transcriptome. (**E**) Gene ontology (GO) analysis to identify Biological Processes in the naïve RPE transcriptome. (**F**) Relative mRNA expression of Gsto1 mRNA in naïve RPE, retina and choroid using qRT-PCR, normalized to 18 S ribosomal RNA (Rn18s). Bar graphs represent mean ± SEM. Statistical analysis was performed using one-way ANOVA with repeated measures followed by Tukey’s post hoc test. *p < 0.05; ****p < 0.0001. (**G**) Immunofluorescence analysis of retinal sections from C57BL6/J mice and human showing cell nuclei (blue), GSTO1 (green), the RPE cell marker RPE65 (red). The right quadrants of each composite image represent the merged overlay of the green, red, and blue channels. Images were acquired using the Leica STELLARIS 8 FALCON Confocal Microscope, 40x objective. Scale bar = 50 μm

Although the SRIRS method to isolate RPE RNA resulted in considerable enrichment of RPE RNA, retinal tissue can carry over into the eyecup preparation. An initial KEGG pathway enrichment analysis of the 2671 genes (TPM ≥ 45) in the Nv mouse RPE preparations showed significant enrichment (FDR = 3.7E-10) of 18 ‘Phototransduction’ KEGG pathway genes, including PR-expressed genes. To correct for this, the Nv RPE transcriptome was compared to that of retina samples obtained from the same naïve eyes. DESeq2 analysis identified 4454 retina DEG with significant (padj ≤ 0.05) 2-fold greater transcript abundance in Nv retina than in Nv RPE. A Venn comparison identified 225 of these retina > RPE DEG as present in the RPE transcriptome (Fig. 1A, Supplemental Data Table S1A); no known RPE marker or signature genes were included in this intersection of sets and the majority represented photoreceptor-specific genes, with the most significantly enriched KEGG pathway being ‘Phototransduction’ (14 genes, FDR = 2.1E-18) and the most significantly enriched GO biological process group being ‘Visual Perception’ (28 genes, FDR = 2.7E-20) (Supplemental Tables S1C and S1D). The 225 genes were therefore excluded from the RPE transcriptome, resulting in mouse RPE transcriptome containing 2446 genes highly expressed mouse RPE genes and representing 11.4% of all genes mapped in Nv RPE samples (Supplemental Data Table S1F). This is considered the mouse RPE transcriptome.

Several previous studies have endeavored to defined RPE signature genes. The 2446-gene mouse RPE transcriptome was compared to three previously published RPE signature gene lists which were derived from: (1) human fetal and adult RPE tissue [36], (2) a mouse laser captured RPE transcriptome compared to human RPE cell data sets [35], and (3) a mouse RPE cell cluster transcriptome derived from single cell sequencing [33]. Venn comparison with a list of 627 combined RPE signature genes from these three publications identified 277 common genes included in the present mouse RPE transcriptome at least one of these previous publications (Fig. 1B, Supplemental Data Table S1G). Comparing to the individual RPE gene lists, 64% (223 of 349) of Lehmann mouse RPE signature genes are in common with the present transcriptome. In contrast, only approximately 30% of the Bennis (83 of 272) and Strunnikova (46 or 154) human RPE signature genes are shared with the present mouse RPE transcriptome (Supplemental Data Table S1G). Importantly, only 14 genes (*Bmp4, Crim1, Degs1, Gja1, Itgav, Mfap3l, Pdpn, Ptgds, Rbp1, Rnf13, Rpe65, Slc4a2, Sulf1* and *Ttr*) are common to the present mouse RPE transcriptome and all three RPE signature gene lists (Fig. 1C). This is not surprising, given that just 19 genes are common to all three published RPE signature gene lists (Supplemental Data Table S1G). The 5 common RPE signature genes that are not within the present RPE transcriptome are: *Sema3c* (TPM = 30.5), *Rragd* (TPM = 19.0), *Cdh3* (TPM = 15.8), *Lhx2* (not mapped) and *Srfp5* (not mapped). Other notable RPE genes that are in one or two of the published RPE signature gene lists but are missing from the present mouse RPE transcriptome are: *Ins2* (TPM = 11.2), *Mertk* (TPM = 9.4) and *Best1* (TPM = 1.3).

### Analysis of the mouse RPE transcriptome

Enrichment analysis was performed with the 2446-gene mouse RPE transcriptome (Fig. 1D and E, Supplemental Table S1H-K). The enriched pathways and GO groups identified well-known features and functions of the RPE. Genes in the KEGG pathways ‘Phagosome’ (45 genes, FDR = 9.1E-06) and ‘Lysosome’ (41 genes, FDR = 4.8E-07) are enriched. In keeping with pigmentation of RPE, the ‘Melanosome’ cellular component GO group genes are highly enriched (56 genes, 5.0E-21, Supplemental Table S1I). Regarding maintenance of outer blood-retinal barrier (oBRB), the transcriptome contains 52 ‘Tight Junction’ related genes (FDR = 5.1E-09), including several claudin genes (*Cldn3, Cldn4, Cldn7* and *Cldn23*) and zonula occludens genes (*Tjp2* and *Tjp3*). Several other claudin mRNAs were mapped, but with TPM < 45. Notably, despite its published importance to RPE barrier function [3], *Cldn19* (TPM = 10.1) did not make it into our mouse RPE transcriptome, nor into any of the published RPE signature gene lists.

Surprisingly, enrichment analysis of the mouse RPE transcriptome revealed several KEGG groups related to ‘Pathways of Neurodegeneration’ (189 genes, FDR = 1.2E-43), which include the most significantly enriched pathway, ‘Parkinson Disease’ (155 genes, FDR = 1.3E-65) (Fig. 1D). This pathway encompasses genes included in other enriched KEGG pathways, including ‘Oxidative Phosphorylation’ (99 genes, FDR = 1.7E-58), ‘Protein Processing in the Endoplasmic Reticulum’ (66 genes, FDR = 1.4E-16), ‘Ubiquitin-mediated Proteolysis’ (41 genes, FDR = 1.2E-05) and ‘Proteosome’ (36 genes, FDR = 9.1E-23). In addition, ‘Ribosome’ genes, encoding large and small ribosomal subunits, are very significantly enriched (102 genes, FDR = 1.3E-60). Similarly, analysis of enrichment of biological process GO groups identified highly significant enrichment of several groups related to ‘Translation’ (209 genes, FDR = 3.3E-36), including the most significantly enriched ‘Peptide Metabolic Process’ (262 genes, FDR = 4.5E-44) (Fig. 1E). In keeping with protein synthesis, enriched cellular component GO groups include many related to ribosomes and their components (Supplemental Data Table S1I) and many enriched GO molecular function groups are related to ribosome structure and function, regulation of mRNA translation and protein processing (Supplemental Data Table S1J). There are also a relatively large number of enriched cellular component genes (377, 7.3E-26) encoding ‘Endoplasmic Reticulum’ proteins (Supplemental Data Table S1I) suggesting that many plasma membrane-targeted or secreted proteins may be produced by mouse RPE.

Many genes in the mouse RPE transcriptome are metabolism-related, with 336 genes in the KEGG ‘Metabolic Pathways’ group (FDR = 1.8E-23) (Fig. 1D). Given that RPE metabolism is highly oxidative to allow utilization of lactate produced by PR and fatty acids derived from PR outer segments [37], it is not surprising that several highly enriched GO biological process groups were also related to ‘Cellular Respiration’ (98 genes, FDR = 2.5E-31), ‘Oxidative Phosphorylation’ (74 genes, FDR = 4.8E-34) and ‘Electron Transport Chain’ (74 genes, FDR = 4.7E-28) (Fig. 1E). Similarly, several highly enrich GO molecular function groups were related to electron transport chain and oxidative phosphorylation (Fig. 1E, Supplemental Data Table S1J). Enriched cellular component GO groups also include several related to mitochondria and respiration (Supplemental Data Table S1I). In keeping with the recent demonstration that peroxisomal β-oxidation is essential for RPE lysosomal function and digestion of very long chain polyunsaturated fatty acids present in PR OS [38], the analysis identified significant enrichment of genes in the KEGG ‘Fatty Acid Degradation’ pathway (21 genes, FDR = 1.5E-06), the GO biological process group ‘Fatty Acid Oxidation’ (34 genes, FDR = 4.9E-05), the KEGG ‘Peroxisome’ pathway (24 genes, FDR = 2.3E-03) and the GO cellular component ‘Peroxisome’ group (34 genes, FDR = 1.8E-03).

Several other highly expressed genes in the mouse RPE transcriptome support established RPE functions. For example, in keeping with the RPE’s apical import of PR-produced lactate and export of β-hydoxybutyrate produced by fatty acid oxidation, *Slc16a1* mRNA, encoding the monocarboxylate transporter MCT1, is relatively highly expressed (TPM = 89.9). Interestingly, the abundance of *Slc16a8* mRNA (TPM = 167.2), encoding MCT3, which is thought to transport excess lactate out of the RPE at the basolateral side [37], exceeds that of *Slc16a1*.

Enrichment analyses also identified the KEGG pathway ‘Glutathione Metabolism’ (FDR = 2.0E-05) and the GO biological process group ‘Glutathione Metabolic Process’ (FDR = 1.1E-05) (Supplemental Table S1G). These groups contain several glutathione peroxidase genes (*Gpx1*, *Gpx3* and *Gpx4*) and several glutathione S-transferase genes (*Gsta1, Gsta2, Gsta3, Gsta4, Gstm1, Gstm2, Gsto1, Gstp1* and *Mgst1*). Remarkably, *Gsto1* (glutathione S-transferase omega 1) has the most mapped sequences in the mouse RPE transcriptome, with a TPM = 24336.4. Although GSTO1 protein expression was reported for a wide range of human cells and tissues [39], in porcine corneal epithelium [40] and mouse cone photoreceptors [41], to the best of our knowledge GSTO1 expression by RPE has not been described. We validated the high expression of *Gsto1* mRNA in naïve RPE using qRT-PCR (Fig. 1F). *Gsto1* mRNA expression in RPE was much higher than in retina (177-fold, *p* < 0.0001) and choroid (3.5-fold, *p*-value < 0.001). Because *Gsto1* was reported to be relatively highly expressed in liver [39, 42], mRNA levels in mouse liver and RPE RNA were also compared. Surprisingly, *Gsto1* mRNA expression in RPE was found to be 811-fold higher in RPE than in liver (*p* < 0.001), when using 18S ribosomal RNA as internal control mRNA (data not shown). Thus, qRT-PCR confirmed the RNA-Seq results suggesting exceptionally high *Gsto1* mRNA expression in the mouse RPE.

Immunofluorescence (IF) in ocular sections, using a gene knockout-validated antibody to GSTO1 protein and a well-characterized antibody to RPE65, showed co-localization, suggesting GSTO1 protein expression in mouse RPE (Fig. 1G). However, the analysis did not detect GSTO1 IF in the cone PR, as previously published [41]. We also found that the anti-GSTO1 antibody co-localized with anti-RPE65 in the human RPE, with no apparent expression in human PR (Fig. 1G). Similarly, in rat GSTO1 IF co-localized with RPE65 and no signal was apparent in PR (Supplement Figure S2). In contrast, although predicted to bind pig GSTO1, the antibody showed minimal co-localization with RPE65 in pig and rhesus monkey sections. Rather, in pig and monkey most GSTO1 IF signal was located basal to the RPE, in regions corresponding to the Bruch’s membrane and the choriocapillaris (Supplement Figure S2).

### Temporal effects of retinal detachment on RPE gene expression

**Fig. 2:**
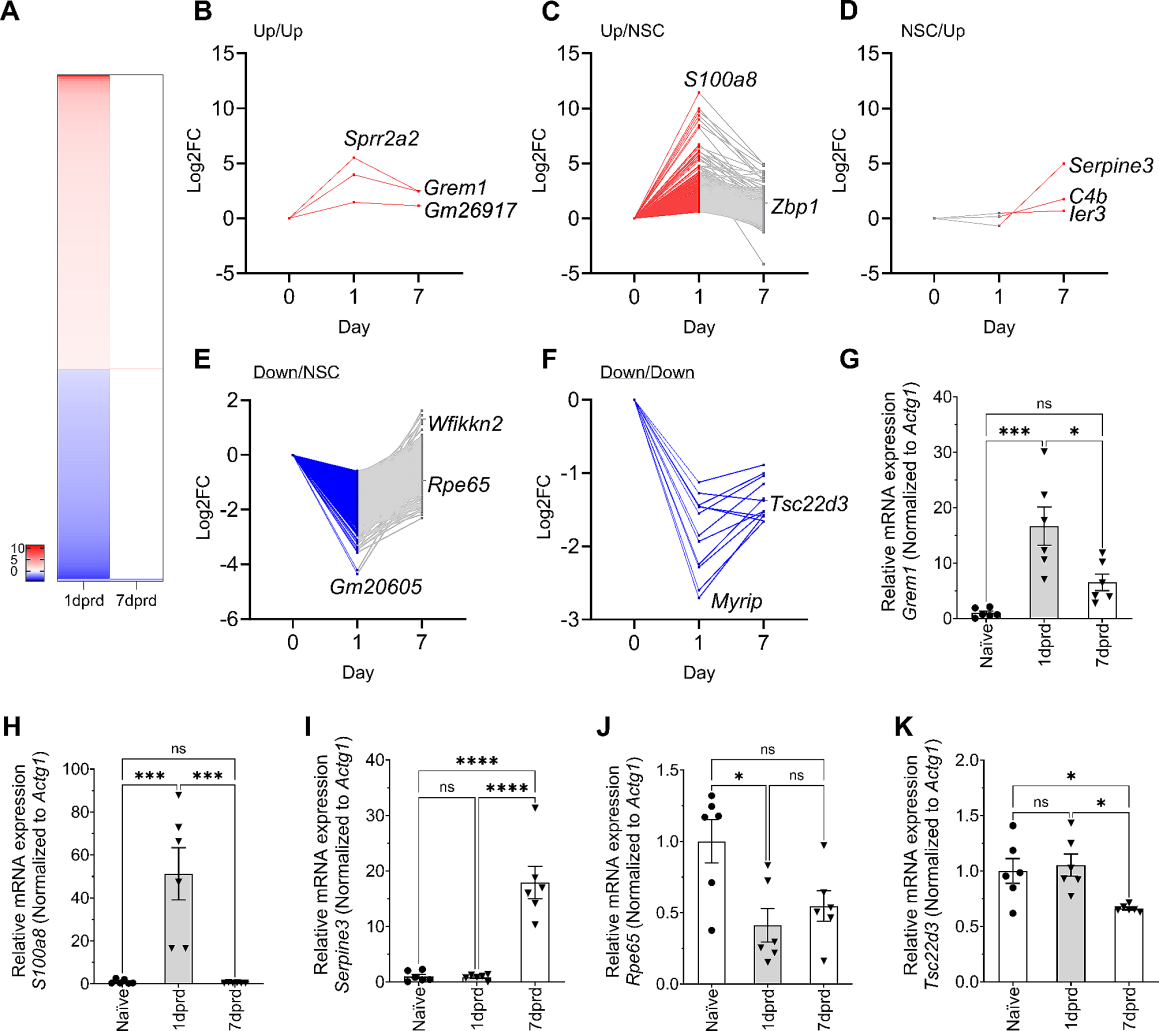
Temporal gene and protein expression changes after retinal detachment and sequencing validation. (**A**) Heatmap showing significantly upregulated (FC ≥ 1.5, p_adj_≤0.05, red) and downregulated (FC ≤ 0.67, p_adj_≤0.05, blue) DEGs 1 and 7 dprd. (**B-F**) Relative expression values of DEG grouped by temporal expression. (**B**) Log2FC of all DEGs significantly upregulated at both 1 and 7 dprd (Up/Up). (**C**) Log2FC of DEGs significantly upregulated only at 1 dprd (Up/NSC). Gray indicates DEG that were not significantly changed (NSC). (**D**) Log2FC of DEGs significantly upregulated only at 7 dprd (NSC/Up). (**E**) Log2FC of DEGs significantly downregulated only at 1 dprd (Down/NSC). (**F**) Log2FC of DEGs significantly downregulated at both 1 and 7 dprd (Down/Down). (**G-K**) QRT-PCR of temporal DEG expression changes after retinal detachment for RNA-Seq validation. Relative mRNA expression of (**G**) *Grem1*, (**H**) *S100a8*, (**I**) *Serpine3*, (**J**) *Rpe65*, and (**K**) *Tsc22d3*, obtained from isolated naïve RPE (*n* = 6), as well RD RPE at 1 dprd (*n* = 6) and 7 dprd (*n* = 6). Bar graphs represent mean ± SEM. Statistical analysis was performed using one-way ANOVA with repeated measures followed by Tukey’s post hoc test. *p < 0.05; **p < 0.01; ***p < 0.001, ****p < 0.0001

A previous study using in situ hybridization found that the mouse RPE exhibits altered levels of mRNAs for several genes after RD at 4 dprd [18]. To comprehensively examine the RPE’s response to detachment, and to define the earlier response, we used RNA sequencing to examine gene expression changes at 1 and 7 dprd. Detachments representing approximately 50% of the retina were produced, and no efforts were made to separate the RPE in the detached region from those still associated with the retina. Thus, the transcriptomes represent a mixture of RPE under RD conditions, both with and without physical association with the neural retina. For transcriptomic comparisons 1 dprd (*n* = 6) and 7 dprd (*n* = 6) RD groups were each compared to a single Nv control group (*n* = 5). DEG were defined as those with a baseMean ≥ 19.5, 0.67 ≥ FC ≥ 1.5 (-0.585 ≥ Log2FC ≥ 0.585) and P_adj_≤0.05, with baseMean defined as the average of normalized counts for all samples, relative to size factors. The analysis showed an extensive acute response to RD at 1 dprd, with 2293 total DEG identified – 1334 upregulated and 959 downregulated DEG. Comparison of 7 dprd group to the Nv group made obvious that these transcriptomic changes had diminished; only 18 significant DEG were identified at 7 dprd, with 6 upregulated and 12 downregulated genes. The DEG groups are shown as organized into those that were significantly upregulated at both times post RD (Up/Up, 3 genes, Fig. 2B), significantly up only at 1 dprd (Up/NSC, 1328 genes, Fig. 2C), significantly up only at 7 dprd (NSC/Up, 3 genes, Fig. 2D), significantly down only at 1 dprd (Down/NSC. 947-genes, Fig. 2E), and significantly downregulated at both times post RD (Down/Down, 12 genes, Fig. 2F).

To validate the RNA-Seq DEG, mRNA expression of genes from each DEG temporal group was tested in a validation set of RNAs obtained from a separate set of detachments using qRT-PCR. *Grem1* mRNA, an example of an Up/Up DEG, was found to be upregulated 17-fold at 1 dprd (*p* ≤ 0.001) and trended up 6.5-fold at 7 dprd (*p* = 0.25) (Fig. 2G). An Up/NSC DEG, *S100a8* mRNA, was increased by 51-fold at 1 dprd (*p* < 0.0001) and not increased at 7 dprd (*p* = 1.0) (Fig. 2H). The NSC/Up DEG *Serpine3* mRNA was not change at 1 dprd (*p* = 0.999) but was significantly increased 18-fold at 7 dprd (*p* < 0.0001) (Fig. 2I), consistent with the RNA-Seq results showing it to be the most highly increased mRNA in the long-detached RPE. However, using the two commercially available antibodies to SERPINE3 that we were able to identify, we were unable to convincingly demonstrate increased SERPINE3 protein expression in the detached RPE (Supplemental Figure S4). The Down/NSC DEG, Rpe65 was significantly downregulated (0.41-fold, *p* < 0.01) at 1 dprd and trended down at 7 dprd (0.55-fold, *p* = 0.08). The Down/Down DEG, Tsc22d3 was found to not be downregulated at 1 dprd (*p* = 0.963), but was significantly downregulated at 7 dprd (0.66-fold, *p* < 0.05). Additional DEG were validated by qRT-PCR in this sample set (Supplemental Figure S3) and largely validated results from RNA-Seq.

**Fig. 3:**
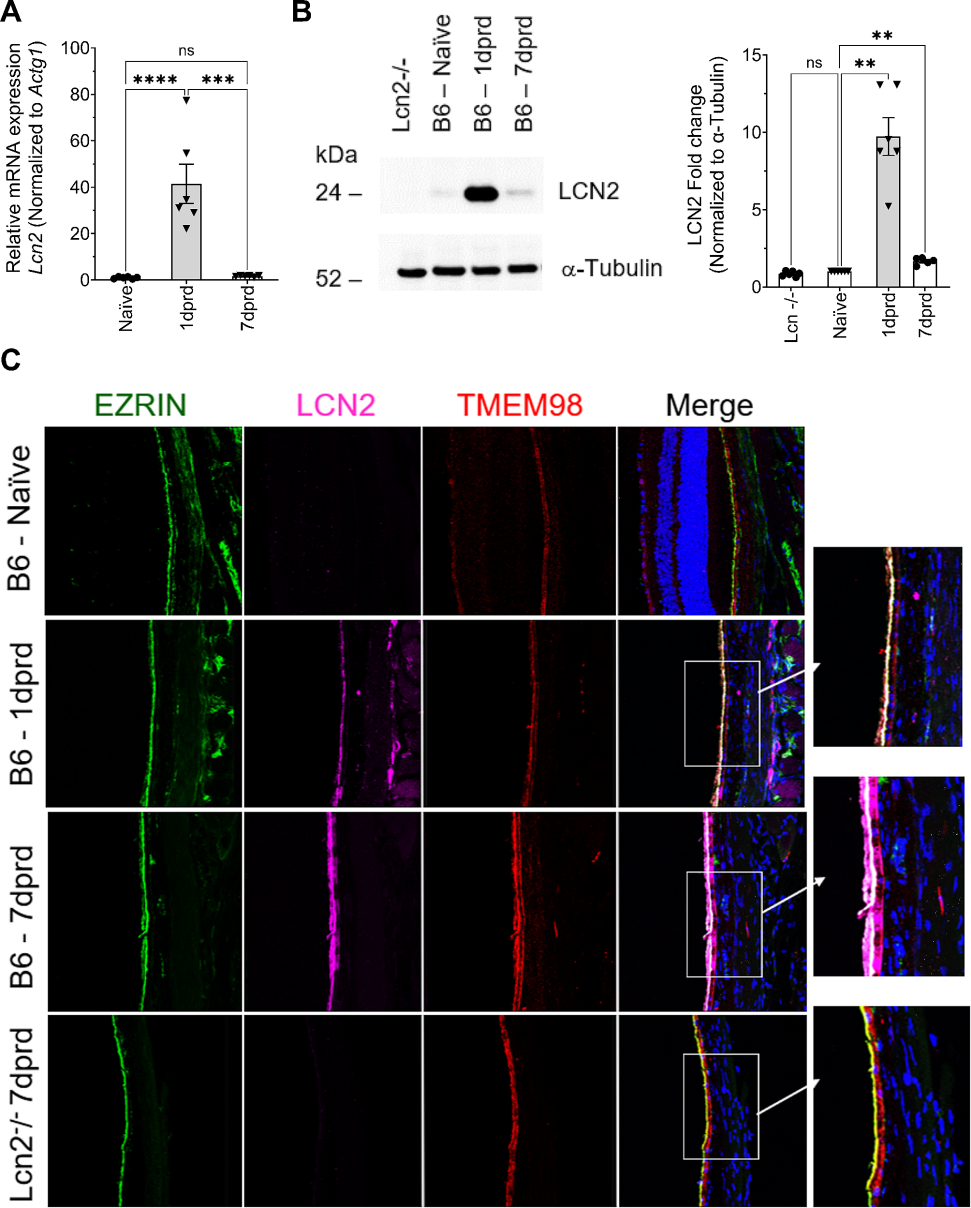
Temporal LCN2 expression changes after retinal detachment for sequencing validation. (**A**) QRT-PCR analysis of *Lcn2* mRNA levels in naïve and detached RPE at 1 dprd and 7 dprd (*n* = 6/group). (**B**) Representative immunoblot of LCN2 protein in RPE/choroid lysates from Lcn2 knockout mice (Lcn2^−/−^), as well as RPE/choroid lysates from wildtype C57BL6/J mice under naïve, 1 dprd and 7 dprd conditions. Graph shows normalized results for 6 independent blots (*n* = 6 mice/group) showing mean ± SEM. Statistical analysis was performed using one-way ANOVA with repeated measures followed by Dunnett’s post hoc test. **p < 0.01. (**C**) IF co-localization of LCN2 (magenta) with Ezrin (green) and TMEM98 (red). Ezrin is an actin binding protein located in the apical microvilli of RPE cells, while TMEM98 is a transmembrane protein located at both the apical and basolateral surfaces of the RPE [46]. Ocular sections from C57BL6/J mice under naïve, 1 dprd and 7 dprd conditions and a section from *Lcn2* KO mice with detached retina at 7 dprd. Staining for cell nuclei (blue) is shown in the merged images (right column) and magnified regions (boxes). Images were acquired using the Leica STELLARIS 8 FALCON Confocal Microscope, 40x objective

Lipocalin-2, which among its many functions can act as a inducer of chemokine expression [43–45], was identified as one of the DEG most highly responsive to RD, with a 42.9-fold increase at 1 dprd in the RNA-Seq analysis. We therefore chose to validate *Lcn2* mRNA results and examine LCN2 protein expression. Samples obtained from a germline Lcn2 KO mouse were used as a negative control. QRT-PCR confirmed a 40-fold increase in *Lcn2* mRNA (*p* < 0.0001), which reverted to normal at 7 dprd (Fig. 3A). Western blotting showed that LCN2 protein in the RPE/choroid was highly increased at 1 dprd versus Nv (9.7-fold, *p* < 0.01), and significantly elevated at 7 dprd (1.7-fold, *p* < 0.01) (Fig. 3B). IF of ocular sections revealed that LCN2 protein was nearly undetectable in naïve B6 mouse ocular sections (Fig. 3C). At 1 dprd, LCN2 protein IF was intense on the apical surface of the RPE, which was confirmed by co-localization with the apical RPE marker Ezrin (Fig. 3C). Interestingly, LCN2 protein IF was even more apparent in RPE at 7 dprd and was also located intracellularly and on the basolateral surface in some regions, as demonstrated by a broader distribution and co-localization with TMEM98, a transmembrane protein located at both the apical and basolateral surfaces of the RPE [46]. We also noted intense LCN2 IF in the retina at 1 and 7 dprd (data not shown). Importantly, antibody binding was highly specific, for sections from the *Lcn2* KO mouse exhibited no anti-LCN2 IF background, even in sections from 1 to 7 dprd (data not shown).

**Fig. 4:**
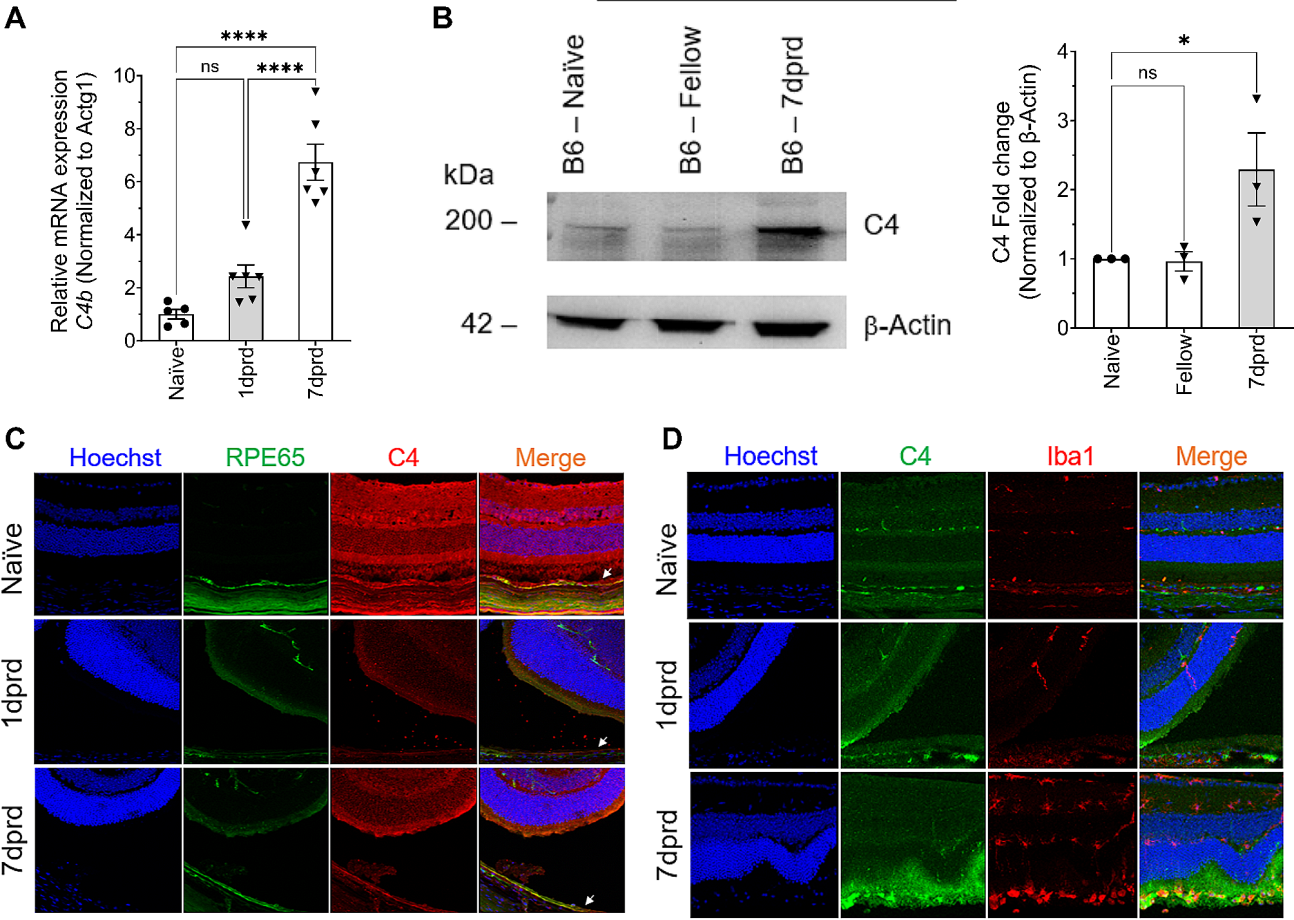
C4 protein localization after retinal detachment for sequencing validation. (**A**) QRT-PCR analysis of *C4b* mRNA levels in naïve and detached RPE at 1 dprd and 7 dprd. (**B**) Representative immunoblot of complement C4 in the soluble fluid fraction from C57BL6/J mouse eyes under naïve, contralateral (fellow at 7 dprd), and detached (7 dprd) conditions with protein samples separated under non-reducing conditions. Blots are representative of 3 independent animals in each condition. Bar graphs represent mean ± SEM. Statistical analysis was performed using one-way ANOVA with repeated measures followed by Dunnett’s post hoc test. *p < 0.05; ****p < 0.0001. (**C**) IF analysis of ocular sections from C57BL6/J mice under naïve, 1 dprd and 7 dprd conditions. Cell nuclei staining (blue), and IF of anti-RPE65 mAb (green) and anti-C4 serum (red) are shown. (**D**) IF analysis of C4 protein in retinal and subretinal Iba-1^+^ immune cells in ocular sections from C57BL6/J mice under naïve, 1 dprd and 7 dprd conditions. IF of anti-C4 mAb (green), anti-Iba1 mAb (red, a microglia and monocyte/macrophage marker [48]). Staining cell nuclei (blue) is shown in the merged images (right column). Images were acquired using the Leica STELLARIS 8 FALCON Confocal Microscope, 40x objective

C4b, which plays an essential role in both classical and lectin complement pathways [47], was one of few DEG upregulated at 7 dprd. A significant (*p* < 0.0001) increase in C4b mRNA of 6.7-fold at 7 dprd was validated by qRT-PCR in a repeat set of samples (Fig. 4A). C4 protein expression was thus examined by western blotting and IF in ocular sections. Western blotting of soluble ocular fluid (vitreous, retinal soluble protein and subretinal fluid) with an often-cited mAb to C4 protein showed no increase at 1 dprd and a significant (*p* < 0.05) 2.3-fold increase at 7 dprd (Fig. 4B). In IF analyses, anti-C4 serum IF was colocalized with anti-RPE65 IF in the RPE, but the serum also produced staining of the neuroretina, even in naïve samples (Fig. 4C). Anti-C4 immunolabeling with the mAb showed intense IF on Iba1^+^ subretinal immune cells (Fig. 4D), suggesting that C4 protein may accumulate on or in microglia and perhaps monocyte-derived macrophages in the subretinal space.

### Analysis of differentially expressed genes after one day of retinal detachment

Previous studies have shown that epithelial barriers can react to injury and infection by taking on an innate immune phenotype to provide local immunity [49]. DESeq2 comparison of RNA-Seq reads from naïve RPE (*n* = 5) and RPE with retinal detachments harvested at 1 dprd (*n* = 6) identified 2297 DEG with a baseMean expression cutoff of 19.5, 0.67 ≥ FC ≥ 1.5 and p_adj_≤0.05 (Supplemental Table S2B). We hypothesized that the RPE would react to RD by rapidly increasing its barrier function and mounting a sterile innate immune response. To test this, we examined DEG upregulated at 1 dprd and used KEGG pathway and GO group enrichment analysis to characterize them. Upregulated DEG were defined as those with FC ≥ 1.5 (Log2FC ≥ 0.585) and P_adj_≤0.05, which yields 1334 upregulated DEG (Supplemental Table S2C). Within this list are 18 genes (1.3%) that are expressed at greater levels in retina than RPE. Because of the small fraction they represent, and because none are genes associated with phototransduction, these 18 DEG were not excluded from the analysis of DEG.

**Fig. 5:**
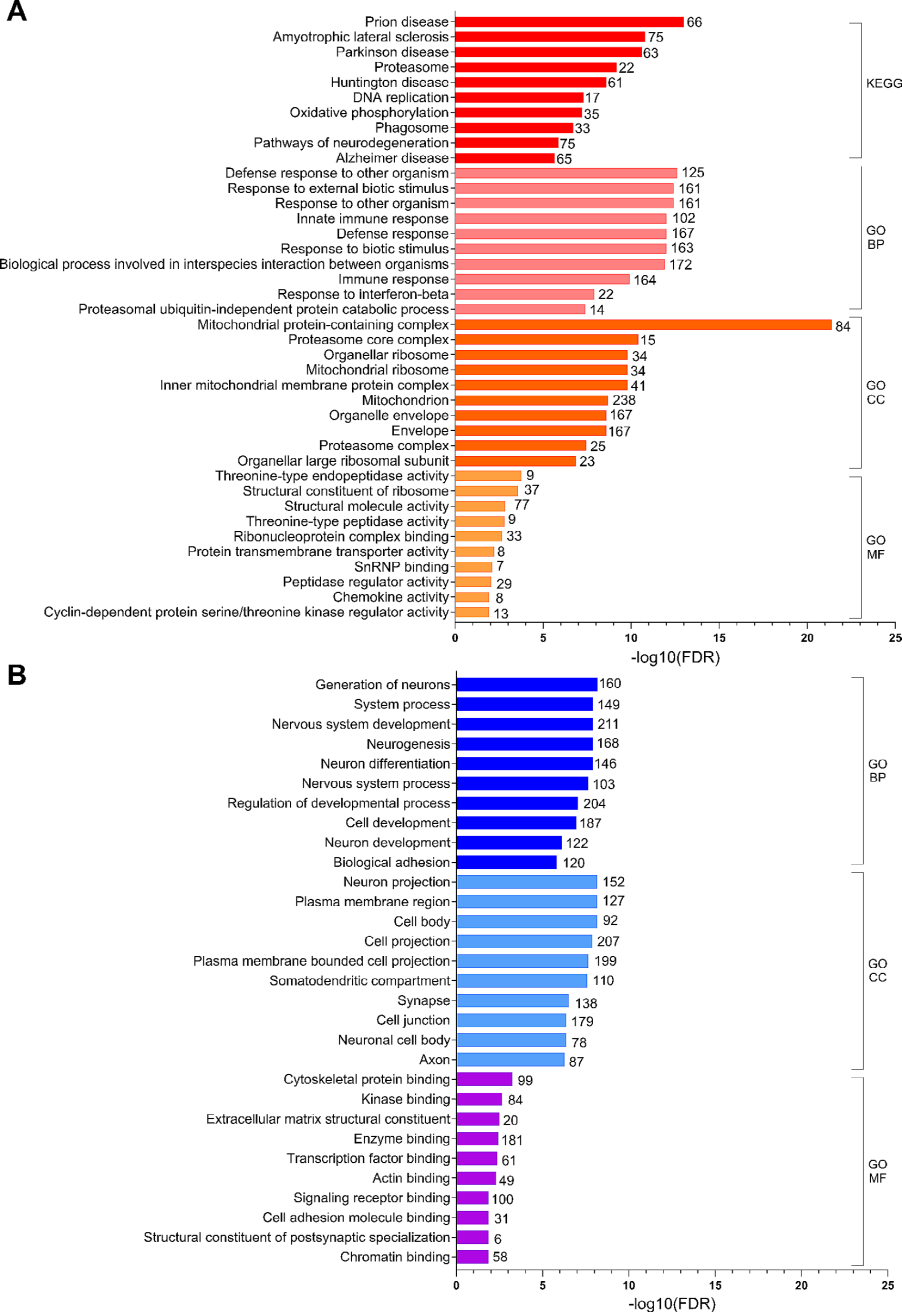
Enrichment analysis of differentially expressed genes one day following retinal detachment. (**A**) Gene enrichment analyses for KEGG pathways (red), gene ontology (GO) biological function groups (BP, salmon), GO cellular component groups (CC, orange) and GO molecular function (MF, yellow) groups for significantly upregulated DEG (FC ≥ 1.5, p_adj_≤0.05) at 1 dprd. (**B**) Gene enrichment analyses for GO biological function groups (BP, blue), GO cellular component groups (CC, light blue), and GO molecular function groups (MF, purple) for significantly downregulated DEG (FC ≤ 0.67, p_adj_≤0.05) at 1 dprd. Y-axis shows the negative log_10_ of the false discovery rate (FDR) for each group. Numbers of significant DEG in each group are indicated at the end of the bars. Note that significantly enriched KEGG pathways were not identified for the downregulated DEG at 1 dprd

KEGG pathway enrichment was examined in the 1334 upregulated DEG at 1dprd. Several neurodegeneration-related groups are significantly enriched, including ‘Prion Disease Amyotrophic Lateral Sclerosis’, ‘Parkinson Disease’, ‘Huntington Disease’, ‘Pathways of Neurodegeneration’ and ‘Alzheimer Disease’ are highly enriched (Fig. 5A). ‘Prion Disease’ is the most significantly enriched KEGG pathway (FDR = 9.0E-14). Although neurodegeneration seems incongruous, it should be noted that, the KEGG neurodegeneration groups are dominated by genes in pathways related to oxidative phosphorylation, mitochondria-initiated apoptosis, ER-stress associated degradation, and proteosome genes (Supplemental Table S2E). Indeed, KEGG groups related to oxidative phosphorylation (‘Oxidative Phosphorylation’, ‘Thermogenesis’, ‘Chemical Carcinogenesis’, ‘Diabetic Cardiomyopathy’, and ‘Non-Alcoholic Fatty Liver Disease’) and phagosome-related groups (‘Phagosome’, ‘Rheumatoid Arthritis’, and ‘Tuberculosis’) are also significantly enriched. Other highly enriched KEGG pathway groups include: a cell cycle group (‘Cell Cycle’ and ‘P53 Signaling Pathway’), a DNA replication/repair group (‘DNA Replication’ and ‘Nucleotide Excision Repair’), a ‘Drug and Pyrimidine Metabolism’ group, and a protein synthesis and secretion group (‘Ribosome’, ‘Protein Export’ and ‘Protein Processing in the Endoplasmic Reticulum’). Of note is that more than 10% of the upregulated DEG (148 genes) are included in the KEGG ‘Metabolic Pathways’ group. Thus, the KEGG enrichment analysis suggests that the RPE mounts a stress response following RD that in many ways resembles those associated with neurodegenerative diseases.

KEGG enrichment analysis also identified pathways associated with infection (‘Influenza A’, ‘Epstein-Barr Virus Infection’, ‘Viral Carcinogenesis’, ‘Measles’, and ‘NOD-like Receptor Signaling’ pathways), as well as ‘Viral Protein Interaction with Cytokine and Cytokine Receptors’, ‘Cytokine/Chemokine Signaling’ and ‘Antigen Processing and Presentation’ pathways (Supplemental Table S2E) suggesting the RPE mounts an innate immune response to retinal detachment. Likewise, analysis of enrichment of biological process GO groups in the upregulated DEG at 1 dprd revealed many groups characterized by innate immune defense responses (Fig. 5A, Supplemental Table S2F). These include the most significantly enriched group, ‘Defense Response to Other Organism’ (FDR = 2.2E-13). Other groups in this category include: ‘Innate Immune Response’, ‘Immune Response’, ‘Response to Cytokine’, and ‘Response to Virus’. In addition, innate immune response groups include ‘Response to Interferon − Beta’, ‘Response to Interferon − Gamma’, and ‘Positive Regulation of Tumor Necrosis Factor Superfamily Cytokine Production’. As with KEGG pathways, several enriched GO biological process groups are also related to cell cycle and cell division, and to protein catabolic processes (Supplemental Table S2F). Thus, as predicted the analysis suggests the RPE’s transcriptional response to RD involves upregulation of genes involved with an innate immune defense response, along with the seemingly contradictory processes of cell growth and protein degradation.

Analysis of cellular component gene ontology groups in the upregulated DEG at 1 dprd (Fig. 5A, Supplemental Table S2G) revealed that the most highly enriched group is ‘Mitochondrial Protein-containing Complex’ (FDR = 4.4E-22). Nearly 18% (238) of the 1334 up-regulated DEG at 1 dprd encode ‘Components of Mitochondrion’ (FDR = 2.1E-09). These include 34 ‘Mitochondrial Ribosomal Subunits’ (FDR = 1.6E-10), which may reflect increased synthesis of electron transport chain proteins that are translated in the mitochondria [50]. Additional enriched cellular component GO groups include 190 gene encoding proteins located to the ‘Endoplasmic Reticulum’ and 25 genes encoding the ‘Proteosome Complex’.

Enrichment of molecular function GO groups upregulated DEG at 1 dprd (Fig. 5A, Supplemental Table S2H) emphasizes the upregulation of proteosome subunits with threonine-type endopeptidase activity, structural components of ribosomes, electron transport proteins, and cytokines with chemoattraction activity (chemokines), including *Ccl2*, *Ccl6*, *Ccl8*, *Ccl9*, *Cxcl4* (Pf4), *Cxcl7* (Ppbp), *Cxcl12* and *Cklf*. One of these chemokine mRNAs, *Ccl8*, was also found to be significantly upregulated by 15-fold (*p* < 0.0001) at 7 dprd in another sample set (Supplemental Figure S3D). In addition, “Protein Transmembrane Transporter Activity’ (FDR = 6.0E-03) genes are enriched, including several mitochondrial membrane translocase genes (Tomm40l, Timm23, Timm22, Tomm22, Timm17b and Tomm20). Genes with ‘SnRNP Binding Function’ are also enriched (FRD = 6.0E-03) and include ‘Splicosomal Small Nuclear Ribonucleoprotein’ genes (Snrpd1, Snrpb2, Snrpc, Snrpd2, Snrpg, Snrpe) and the gene encoding serine/threonine kinase receptor associated protein (STRAP), which enables snRNP RNA binding activity [51]. Also of note is the significant enrichment of genes with ‘Oxidoreductase Activity’ and ‘Glutathione Peroxidase Activity’, including Gpx1, Gpx2, Gpx4, Gpx8, as well as 2 glutathione S-transferase genes, Gstt2 and Mgst1.

### Analysis of downregulated DEG at 1 dprd

Downregulated DEG were defined as those with a baseMean ≥ 19.5, FC ≤ 0.67 and P_adj_≤0.05. This yielded 963 downregulated DEG at 1dprd (Supplemental Table S2I). This set includes 29 genes (3%) that were identified as being expressed higher in retina than RPE. These genes include 3 PR genes, *Reep6* (receptor accessory protein 6), *Slc24a1* (retinal rod Na+/Ca+/K + exchanger), and Stargardt disease gene, *Abca4*. However, Lehmann and coworkers found that *Slc24a1* is highly expressed by the mouse RPE cluster [33], and expression of *Abca4* by RPE is now established [52]. Many highly expressed PR genes, including *Gnat1*, *Pde6g* and *Rho*, were nominally, but not significantly, downregulated in the RPE samples at 1 dprd, which could reflect the decreased association of detached RPE with PR OS.

Downregulated DEG at 1 dprd were analyzed for KEGG pathway and GO group enrichment. No KEGG pathways were significantly enriched in the 1 dprd downregulated DEG list. Analysis of enrichment of biological process GO groups in the downregulated DEG at 1 dprd (Fig. 5B, Supplemental Table S2K) revealed many groups characterized by neurogenesis, including the most significantly enriched group, ‘Generation of Neurons’ (160 DEG, FDR = 6.6E-09) and the group containing the most genes, ‘Nervous System Development’ (211 DEG, FDR = 1.2E-08). Other biological process GO groups are closely related to ‘Synaptic Signaling’ (74 DEG, FDR = 1.4E-05), ‘Cell Adhesion’ (119 DEG, FDR = 1.5E-06), ‘Cell Migration (74 DEG, FDR = 2.6E-05), ‘Cell Morphogenesis’ (104 DEG, FDR = 3.3E-05), and “Cell-Cell Signaling’ (127 DEG, FDR = 1.2E-05). Cell component GO enrichment groups of downregulated DEG at 1dprd are dominated by groups related to ‘Neuron Projection’ (152 DEG, FDR = 7.0E-09), including ‘Axon’ (87 DEG, FDR = 5.4E-07) and ‘Dendrite’ (78 DEG, FDR = 1.2E-05) (Fig. 5B, Supplemental Table S2L). There are also several groups related to ‘Synapse’ (138 DEG, FDR = 3.0E-07). Other groups are related to ‘Cell Junction’ (179 DEG, FDR = 4.3E-07), ‘Basement Membrane’ (21 DEG, FDR = 1.1E-4) and the ‘Apical Part of Cell’ (45 DEG, FDR = 2.0E-3). Enriched molecular function GO groups included several DEG groups related to synapse and extracellular matrix formation and binding (Fig. 5B, Supplemental Table S2M). ‘Signaling Receptor Binding’ (100 DEG, FDR = 4.4E-03) includes many downregulated DEGs encoding growth factors (Ngf, Vwf, Fgf10, Vegfa, Fgf1, and Igf2), as well as numerous cell surface receptors for various ligands. In addition, several groups of DEG related epigenome modification and gene transcription are enriched, including ‘Transcription Factor Binding’ (61 DEG, FDR = 4.4E-03) and ‘Chromatin Binding’ (58 DEG, FDR = 1.4E-02). Also notable is that the genes encoding all 3 natriuretic peptide receptors (Npr3, Npr1 and Npr2) are downregulated DEG at 1 dprd.

The set of downregulated DEG and 1 dprd includes many notable RPE genes, including *Abca4, Bmp4*, *Crim1*, *Hmgcs2*, *Lrat*, *Ptgds*, *Rdh10*, *Rlbp1, Rgr*, *Rpe65*, *Slc4a5*, and *Trpm3*, which all exhibit more than 4-fold downregulation (< 0.25-fold compared to Nv control group level), *Ezr* (0.51-fold versus Nv) and *Ald3a1* (0.60-fold versus Nv). Notably, a majority (165) of the 277 genes common to the mouse RPE transcriptome and one or more published RPE signature gene list trended down at 1 dprd, with 43 being significant downregulated DEG (Supplemental Figure S5). These include *Ald3a1*, *Bmp4*, *Crim1*, *Ezr*, *Hmgcs2*, *Lrat*, *Ptgds*, *Rdh10*, *Rgr, Rlbp1, Rpe65*, *Slc4a5* and *Trpm3*. Of the 112 RPE signature genes that trended up, 38 were significantly upregulated DEG. A large portion of these genes encode mitochondrial proteins, including several oxidative phosphorylation genes, *Atp5g3*, *Atp5j*, *Atp5k*, *Atp5l*, *Atp6v0b*, *Cox7a2*, *Cox7c*, *Ndufa4*, *Ndufb2*, *Ndufc1*, *Ndufs4* and *Uqcrq*.

**Fig. 6:**
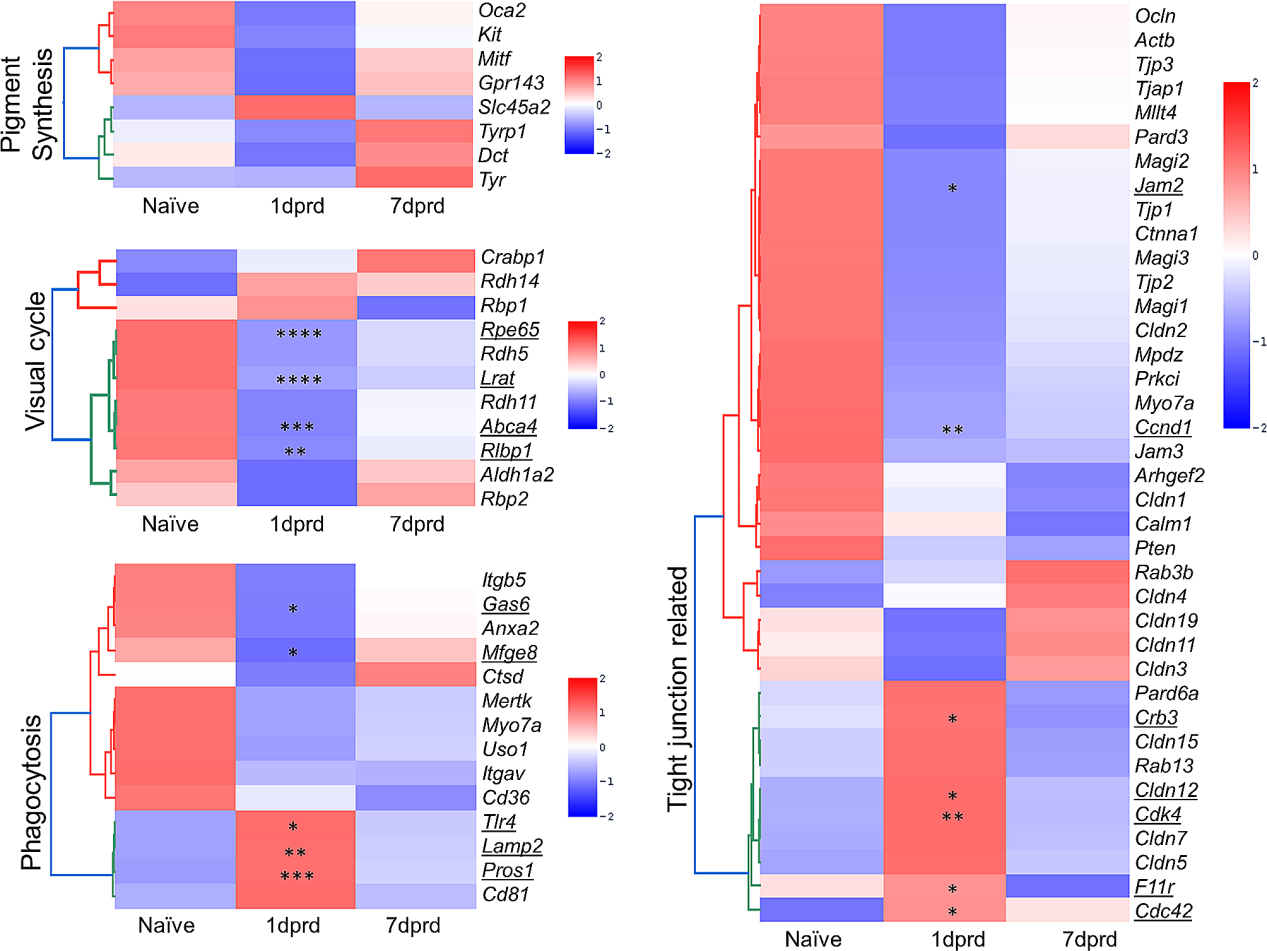
Effects of Retinal Detachment on RPE Functional Genes. Heatmaps showing Z-scores of TPM from RNA-Seq of naïve and detached RPE at 1 dprd and 7 dprd for RPE functional gene sets established by Zhang and coworkers [53]. (**A**) Melanosome/pigment synthesis genes, (**B**) visual phototransduction genes, (**C**) phagocytic genes, and (**D**) tight junction related genes and regulators. Underlined gene names indicate p_adj_≤0.05, asterisks in boxes represent significant p-adjusted values in comparison to naïve group; *p < 0.05; **p < 0.01; ***p < 0.001, ****p < 0.0001

We also examined the Z-scores of expression for RPE functional gene sets established by Zhang and coworkers [53] to examine development of human RPE. These included melanosome/pigment synthesis genes, visual phototransduction genes, phagocytic genes, and tight junction genes and their regulators. The analysis showed that the majority of these RPE genes tended to be downregulated at 1 dprd (Fig. 6). Notably, several visual cycle genes (*Abca4, Lrat*, *Rlpb1* and *Rpe65*), phagocytosis related genes (*Gas6* and *Mfge8*) and tight junction-related genes (*Ccnd1* and *Jam2*) were significantly downregulated at 1 dprd. Some RPE function genes related to phagocytosis (*Lamp2, Pros1* and *Tlr4*) and tight junctions and their regulators (*Cdc42*, *Cdk4*, *Cldn12*, *Crb3* and *F11r*) were significantly upregulated at 1 dprd.

### DEG at 7 dprd

At 7 dprd most transcriptomic changes noted at 1 dprd had diminished, with only 18 significant DEG – 6 upregulated and 12 downregulated genes. Three of the upregulated genes (*Gm26917, Grem1*, and *Sprr2a2*) were also significantly increased at 1 dprd, while the other 3 (*C4b, Ier3* and *Serpine3*) were not (Fig. 2). We validated the upregulation of *Serpine3* and *C4b* mRNAs at 7 dprd in a repeat experiment and using qRT-PCR (Figs. 2I and 4A). In addition, qRT-PCR validation studies found that mRNAs of the complement factor *C1qa* and the chemokine *Ccl8* were significantly increased at 7 dprd (Figure S3D and S3E), even though these were not significant DEG at 7 dprd in the RNA-Seq analysis.

Expression of all 12 of the DEG downregulated at 7 dprd (*Caskin1, Cdr2, Dusp4, Enpp2, Hmgcs2, Inpp5k, Myrip, Npr1, Olfm1, Paqr9, Shisal1, and Tsc22d3*) was also significantly decreased at 1 dprd in the RNA-Seq analysis. Four of these, *Cdr2*, *Enpp2*, *Hmgcs2*, and *Inpp5k* are among the common 277 RPE signature genes. As noted, significant downregulation of *Tsc22d3* mRNA at 7 dprd was validated, but it was not found to be downregulated at 1 dprd (Fig. 2K). Also, Myrip mRNA trended down in RD RPE at both times, but was not significantly different than naïve (*p* = 0.7158 and *p* = 0.4755, Supplemental Figure S3J).

## Discussion

We used a previously developed method of simultaneously isolating RPE cells from the eyecup and stabilizing their RNA along with RNA sequencing to examine how the RPE responds to separation from the neural retina. Because the effects of RD on contralateral eye are undefined, as the control we used naïve RPE harvested from strain-, sex- and aged-matched mice harvested at the same time of day as the RD RPE. This provided an opportunity to examine the normal mouse RPE transcriptome in some detail. We defined the RPE transcriptome as the 12.5% most highly expressed mRNAs. This strategy was previously used with a cutoff of the top 10% of genes [33]. Some conspicuous retinal genes, mainly those highly expressed in photoreceptors, were present in the RPE transcriptome. This contamination could be unavoidable due to the close association of RPE with PR OS, or could be due to small amounts of retinal tissue left behind in the eye cup preparations. We obtained the transcriptomes for the retinas removed from the same naïve eyes used for RPE RNA preps and did a differential expression analysis between retina and RPE samples. This allowed us to identify 225 DEG in the original RPE transcriptome (mean TPM ≥ 45) that were significantly more than 2-fold higher expressed in retina than RPE. These 225 were highly enriched in genes related to phototransduction, including rhodopsin (Supplemental Data Table S1F), so we removed them from the mouse RPE transcriptome, leaving 2446 genes, 11.4% of mapped genes, for our analysis.

The present mouse RNA transcriptome was compared to three previously defined lists of RPE signature genes [33, 35, 36]. Of the 627 combined RPE signature genes from these three papers, 277 were included in the present mouse RPE transcriptome. The intersection of RPE signature gene lists from the three previous studies includes only 19 common genes, 14 of which are also in the present mouse RPE transcriptome. These 14 genes (*Bmp4*, *Crim1*, *Degs1*, *Gja1*, *Itgav*, *Mfap3l*, *Pdpn*, *Ptgds*, *Rbp1*, *Rnf13*, *Rpe65*, *Slc4a2*, *Sulf1 and Ttr*) could be considered a core RPE signature gene set that is applicable across both rodent and human. Previous common RPE signature genes that were not within the present RPE transcriptome include, *Cdh3*, *Lhx2*, *Rragd* and *Sema3c*, which were mapped, but with TPM < 45.

Other notable RPE signature genes that are in one or two of the published RPE signature gene lists but not within the present 2446-gene mouse RPE transcriptome are: *Best1, Ins2* and *Mertk*. Despite the well-documented necessity of bestrophin-1 for RPE function [54], *Best1* mRNA exhibited relatively low expression in the mouse RPE (TPM = 1.3). However, *Best1* mRNA was significantly more than 3-fold greater in RPE than whole retina. RPE *Ins2* expression was recently found to be induced by phagocytosis, with RPE *Ins2*-derived insulin being necessary for retinal glucose metabolism and PR survival [55]. Mer tyrosine kinase expression is vital for RPE phagocytosis of OS and maintenance of PR health [56]. Likewise, mRNAs for the other TAM receptors, *Tyro3* and *Axl*, were not expressed at high enough levels to make it into our mouse RPE transcriptome. However, differential comparison of Nv RPE and Nv retina transcriptomes found all three of these TAM receptor mRNAs to be significantly enriched by more than 2-fold in the RPE versus retina (data not shown).

The mouse RPE transcriptome included many other genes important for PR OS phagocytosis. Regarding bridging proteins that bind to TAM receptors, the mouse RPE transcriptome included the *Gas6* mRNA, but not *Pros1*, encoding protein S. Interestingly, *Gas6* expression was significantly downregulated and *Pros1* expression significantly upregulated at 1 dprd. In addition, *Itgav* and *Itgb5* mRNAs, encoding components of integrin αvβ5, were included in the RPE transcriptome. Integrin αvβ5 is essential for initial recognition of PR OS by RPE [37]. Also, *Mfge8* mRNA, encoding milk fat globule-epidermal growth factor, was relatively highly expressed by mouse RPE (mean TPM = 142.4). This is in keeping with its role as a αvβ5 bridging protein, which is also essential for RPE PR OS phagocytosis [57]. In contrast, mRNA encoding the scavenger receptor CD36, which thought to stimulate OS internalization [58], was not included in the mouse RPE transcriptome. In addition, the mouse RPE transcriptome contains many other phagocytosis-related and lysosomal genes, and genes that encode most of the proteins known to be involved in RPE phagolysosome maturation [37] are included in the transcriptome. These include mRNAs encoding the small GTPase RAC1 (*Rac1*), Annexin A2 (*Anxa2*), RAB5 (*Rab5b* and *Rab5c*), RAB7 (*Rab7*), and numerous subunits of the vacuolar ATPase H + Transporting V0 that acidifies phagolysosomes (*Atp6v0a1*, *Atp6v0b*, *Atp6v0c*, *Atp6v0d1* and *Atp6v0e*).

Thus, enrichment analyses of the mouse RPE transcriptome identified expected features and functions, such as pigmentation, phagocytosis, lysosomal and proteasomal degradation of proteins, and barrier function. The analyses also revealed enrichment of mitochondrial genes involved in respiration, as well as genes related to protein synthesis and protein processing in the endoplasmic reticulum. A majority of the 277 genes common to the RPE transcriptome and one or more published RPE signature gene sets, as well as genes in the RPE functional gene lists used by Zhang and coworkers [53] to examine development of human RPE, were downregulated at 1 dprd. These include melanosome/pigment synthesis genes, visual phototransduction genes, phagocytic genes, and some tight junction genes and their regulators. The analysis suggests that RPE functions may be compromised after RD. This is consistent with an earlier study of RPE/choroid tissues isolated from cynomolgus monkey eyes after RD, which found the detached RPE quickly loses pigmentation and barrier functions, but recovers these functions after a week of detachment [59].

The analysis of DEG upregulated at 1 dprd suggests that, as predicted, the RPE undergoes a transcriptional shift that includes an innate immune defense response after detachment. This response is accompanied by increased expression of genes associated with oxidative phosphorylation. cell division, protein degradation, mRNA splicing and endoplasmic reticulum protein processing. However, no evidence was obtained to confirm the prediction that the RPE increases its barrier function after RD, in that the upregulated DEGs did not suggest an increase in barrier function and the heatmap examination of tight junction-related genes and regulators established by Zhang and coworkers [53] showed more decrease of expression than increase (Fig. 6). As noted above, Tsuboi and co-workers [59] found that the RPE/choroid lost barrier function temporarily loses barrier function after RD in a monkey model.

The RPE innate immune response to RD includes increased expression of several chemokine mRNAs. We and others have shown that retinal expression of cytokines is increased after RD and that cytokine levels are increased in the vitreous of RD patients [11, 12, 60]. However, mechanistic studies have mostly addressed the inflammatory response of the detached retina, and glial and immune cells within the retina, rather than the response of the RPE. Our study suggests that the RPE may contribute to the overall inflammatory response and may produce chemokines to attract immune cells to the subretinal space after RD. This potential role of the RPE needs to be further tested.

Interestingly, Lcn2, which has been implicated in induction of cytokine expression [43–45], was also very highly upregulated in the detached RPE. Greatly increased expression of Lcn2 mRNA in the detached RPE at 1 dprd was confirmed. Increased LCN2 protein was located on the apical surface of the RPE at 1 dprd and then also within the RPE at 7 dprd. However, because LCN2 is secreted, binds to receptors on the cell surface and is transferred into cells by receptor-mediated endocytosis [61], the origin of the LCN2 protein on and within the detached RPE is uncertain.

LCN2 is a multifunctinal protein that binds iron and facilitates iron transport into and out of cells and acts as a bacteriocide by sequestering iron (reviewed in [61]). LCN2 is also induced by inflammatory cytokines, and its expression is greatly increased in inflammatory diseases and by acute injury. In the eye, LCN2 protein was found to be significantly elevated by nearly 8-fold in the vitreous of RRD patients compared to vitrectomy patients with idiopathic epiretinal membrane, vitreomacular traction syndrome or full-thickness macular hole [62]. LCN2 has been associated with several other retinal diseases, including AMD [63], but a role of LCN2 expression by the RPE, versus the retina and invading neutrophils, is not clear. Recently, Gupta and co-workers provided evidence that increased LCN2 in RPE cells inhibits autophagy and deregulates iron homeostasis, thus promoting inflammasome activation, oxidative stress and ferroptosis. These authors also showed increased expression of LCN2 protein in RPE from the *Cryba1* conditional knockout mouse model of dry AMD and from a small number (*n* = 3) of AMD patients, suggesting that LCN2 may contribute to RPE degeneration in AMD. In contrast, an earlier study found that LCN2 overexpression protected RPE cells from death induced by lipopolysaccharide and hydrogen peroxide treatments [64]. Importantly, we have not found any evidence that RPE degeneration occurs in our mouse model of RD. Whether LCN2 upregulation in the RPE contributes to inflammation, and whether increased LCN2 is protective or detrimental following RD must be determined.

Comparison of upregulated DEG at 1 dprd to the database ‘Co-expression Literature’ [65] revealed highly significant enrichment (FDR = 8.7E-39) of 72 of the 182 genes that were identified by Rattner and co-workers as being increased by more than 2-fold in the mouse RPE at 24 h after light damage (LD) (see supplemental data table in [18]). Thirty-four of the 72 common genes fall under the ‘Defense response’ and ‘Immune Response’ biological process GO groups. The common DEG included chemokines *Ccl6* and *Ccl9*, as well as *Lcn2*. Using in situ hybridization, Rattner and co-workers also examined the effects of RD on changes in gene expression in the RPE at 4 dprd, and observed increased hybridization to *Mmp3*, *Osmr* and *Serpina3n* mRNAs and decreased hybridization to *Rpe65* and *Rdh10* mRNAs [18]. This is in complete agreement with our RNA-Seq results at 1 dprd using different methods of detachment and RPE RNA isolation. Notably, Rattner and co-workers used saline to cause relatively small retinal detachments, whereas in the present study hyaluronic acid polymer solution was used to create relatively large detachments. The previous study removed RPE cells from the eyecup using calcium-free PBS prior to RNA purification, rather than isolating RPE RNA with by the SRIRS method. Importantly, Rattner and coworkers [18] concluded that LD and RD led to similar RPE responses. From the results of ex vivo eyecup culture experiments, they found that the RPE must remain in the presence of the retina for at least 12 h following LD for the RPE to exhibit gene expression changes at 24 h after LD. They inferred that signals from the retina may be necessary for the RPE response to LD. Future studies will test if the RPE’s acute response to RD is due to intrinsic mechanisms in the RPE or extrinsic factors originating in the retina.

The transcriptomic analysis demonstrated that the RPE’s response to RD is initially very intense, but transient. Only 6 upregulated and 12 downregulated DEG were identified at 7 dprd, in contrast to 1334 upregulated and 959 downregulated DEG at 1 dprd. Three of the upregulated genes at 7 dprd, *Gm26917*, *Grem1* and *Sprr2a2*, were also significantly increased at 1 dprd. *Gm26917* encodes a long non-coding RNA (lncRNA) that promotes NF-κB activation in hepatic macrophages and thus stimulates liver inflammation in response to lipopolysaccharide treatment [66]. *Grem1* encodes Gremlin-1, a bone morphogeneic protein antagonist that inhibits RPE differentiation and promotes RPE epithelial-mesenchymal transition [67]. *Sprr2a2* encodes a small proline-rich protein (SPRR) with bacteriocidal activity that is vital for cutanous barrier defence [68]. The role of these DEG in the sustained reponse of the RPE to RD is yet to be tested.

The other 3 upregulated DEG at 7 dprd, *C4b*, *Ier3* and *Serpine3*, were not significantly upregulated at 1 dprd, suggesting that they may be unique to the RPE’s eventual adaptation to RD. *C4b* encodes the complement protein C4, which is necessary for both the classical and mannose-binding lectin complement activation pathways. Increased levels of C4 protein was found in a soluble eyecup fraction (vitreous, soluble retina proteins and subretinal fluid), and a well-characterized anti-C4 mAb indicated that C4 protein accumulated in the subretinal space on PR OS and on or in immune cells attracted to the subretinal space at 7 dprd. It was previously observed that mouse RPE express C4 [69, 70]. The C4 cleavage fragment C4b (not to be confused with the mouse *C4b* gene) can contribute to opsinization of OS, and *C4b* knockeout partially inhibits PR loss in the mouse sodium iodate (NaIO_3_) model of retinal degeneration [71]. The present results suggest that C4 is produced by RPE after RD, and could contribute to the opsinization of orphaned OS in the subretinal space. *Ier3* encodes the immediate early response gene 3 (a.k.a. immediate early response gene X-1), which is expressed in response to DNA damage and other cell stresses. Unlike other immediate early response gene products, IER3 protein lacks a DNA binding element. Mostly studied in cancer cells, several functions have been attributed to IER3, including effects on apoptosis, inflammation and regulation of mitochondrial respiration [72, 73]. *Serpine3* was most highly upregulated DEG at 7 dprd. SERPINE3 is a functionally uncharacterized member of the large family of SERPIN protease inhibitors [74, 75]. Recently the *Serpine3* gene was found to be preferentially lost in species that do not rely on vision, suggesting that *Serpine3* is essential for the development or maintenance of visual function [76, 77]. However, there is a lack of available antibodies to mouse SERPINE3 protein, and we were unable to obtain conclusive evidence of SERPINE3 protein upregulation in the RPE after RD (Supplemental Data Figure S4).

Only 12 DEG were significantly downregulated at both 1 and 7 dprd. These represent 1.25% of the 959 DEG downregulated at 1 dprd. Most of these genes have not been studied in RPE. However, *Enpp2*, encoding the secreted enzyme, ectonucleotide pyrophosphatase/phosphodiesterase 2, was decreased by approximately 64% at 1 dprd and 67% at 1 dprd. ENPP2 is expressed by human induced pluripotent stem cell (iPSC)-derived RPE and ARPE-19 cells and is suspected to contribute to the extracellular production of ADP from ATP [78, 79]. ENPP2 is also known as autotaxin (ATX), and acts as a phospholipase converting lysophosphatidylcholine to the signaling molecule lysophosphatidic acid (LPA) [80]. Thus, sustained downregulation of ENPP2 expression could reduce both ADP and LPA levels in the subretinal space after RD. Another notable gene downregulated at 7 dprd is *Hmgcs2*, encoding 3-hydroxy-3-methylglutaryl-CoA synthase 2. HMGCS2 is a mitochondrial enzyme that catalyzes the first irreversible step in ketogenesis, the condensation reaction between acetyl-CoA and acetoacetyl-CoA to form HMG-CoA. The RNA-Seq analysis showed that Hmgcs2 mRNA is downregulated by 72% and 55% at 1 and 7 dprd, respectively. Previous studies have suggested that the RPE utilizes fatty acids derived PR OS digestion to produce the ketone body β-hydroxybutyrate that is, in turn, transferred back to the retina for consumption by PR [81, 82]. Given that HMGCS2 catalyzes the rate limiting step in ketogenesis, downregulation of its expression after RD could reflect adaptation to reduced phagocytosis and processing of OS membrane lipids by the separated RPE.

A major limitation of the present study is a lack of functional validations. For example, measurement of RPE layer permeability would be required to functionally test the hypothesis that RPE barrier function changes in response to RD. An additional limitation of this study is the use of only male mice. Studies to determine if in female mice RPE exhibit a qualitatively or quantitatively different response to RD are warranted. Another caveat is the use of Healon sodium hyaluronate solution for subretinal injection to cause RD. Healon^®^ PRO is a viscoelastic long polymer form of HA intended for human intraocular injection. Injection of HA polymer solution has been the most often used method of creating experimental retinal detachments for over 30 years (reviewed in [7]). A major reason for using HA polymer solution is the relative permanence of the detachments it produces. High molecular weight HA polymers are used in many medical applications because of their physical properties, biocompatibility and nontoxicity. However, HA can bind to various cell surface receptors and low molecular weight HA polymer fragments may exert biological effects, including immune responses [83–85]. On the other hand, binding of high molecular weight HA polymer to its cognate receptor, CD44, can inhibit inflammatory hyperalgesia [86]. Comparison of the initial RPE (and retinal) responses to RD caused by subretinal injection of HA polymer solution and saline carrier seems warranted. However, the similarity of results for a limited number of RPE genes after saline-mediated RD published by Rattner and coworkers [18] suggests that the use of Healon is not driving the initial RPE response at 1 dprd. On the other hand, it is possible that degradation of Healon in the subretinal space by hyaluronidases or reactive oxygen species could produce low molecular weight HA fragments that affect the RPE response over time. In addition, complement factor C1q can bind to HA [87], which could theoretically alter opsonization of PR OS in the subretinal space after Healon-mediated RD. Because detachments caused by saline injection are relatively short lasting, comparison to another biocompatible polymer, such as carboxymethylcellulose [88], would be required to test the influence of Healon after longer durations of RD.

## Conclusions

Herein, a transcriptome for naïve C57BL/6J mouse RPE was defined, confirming many known RPE functions and revealing novel insights into RPE physiology. Comparison to RPE transcriptomes after RD indicated that the RPE undergoes dramatic phenotypic alterations by the first day post-RD. The identified DEGs not only revealed a transient downregulation of genes related to RPE functions, but also highlighted a robust innate immune response, with notable upregulation of *Lcn2* and several chemokines, suggesting that the RPE may play a role in attraction of immune cells to the subretinal space created by RD. Interestingly, the RPE transcriptome at 7 dprd indicates a return to a relatively normal state, emphasizing the transient nature of the response and a possible adaptation of the RPE to RD. The upregulation of complement gene *C4b* at 7 dprd and its detection in/on subretinal immune cells underscore the potential involvement of the RPE in the inflammatory response to RD, suggesting a role in opsonization of orphaned outer segments that are separated from the RPE. Overall, our findings provide valuable insights into the dynamic interplay between the RPE and immune responses following RD, paving the way for further understanding of the RPE’s contribution to this pathology.

### Electronic supplementary material

Below is the link to the electronic supplementary material.

Supplementary Material 1

Supplementary Material 2

Supplementary Material 3

Supplementary Material 4

## Data Availability

Results of RNA-Seq DEG analysis, VENN comparisons, and DEG KEGG pathway and GO groups enrichment analyses can be found in supplemental data tables accompanying this manuscript. RNA-Seq data are publicly available at GEO (geo@ncbi.nlm.nih.gov) under accession number (GSE261021).
