# Supplementary material for: The mouse retinal pigment epithelium mounts an innate immune defense response following retinal detachment
Source: J Neuroinflammation. 2024 Mar 25;21:74. doi: 10.1186/s12974-024-03062-2 (PMC10964713; doi:10.1186/s12974-024-03062-2)
Supplement: Supplementary file 3 — Supplementary Material 3 [file 12974_2024_3062_MOESM3_ESM.pdf]

Supplemental Figures for:

The mouse retinal pigment epithelium mounts an innate immune defense response following retinal detachment.

Steven F Abcouwer*, Bruna Miglioranza Scavuzzi, Phillip E. Kish, Dejuan Kong, Sumathi Shanmugam, Xuan An Le, Jingyu Yao, Heather Hager and David N Zacks

Department of Ophthalmology and Visual Sciences, University of Michigan Medicine, Kellogg Eye Center, Ann Arbor, MI, 48105, USA

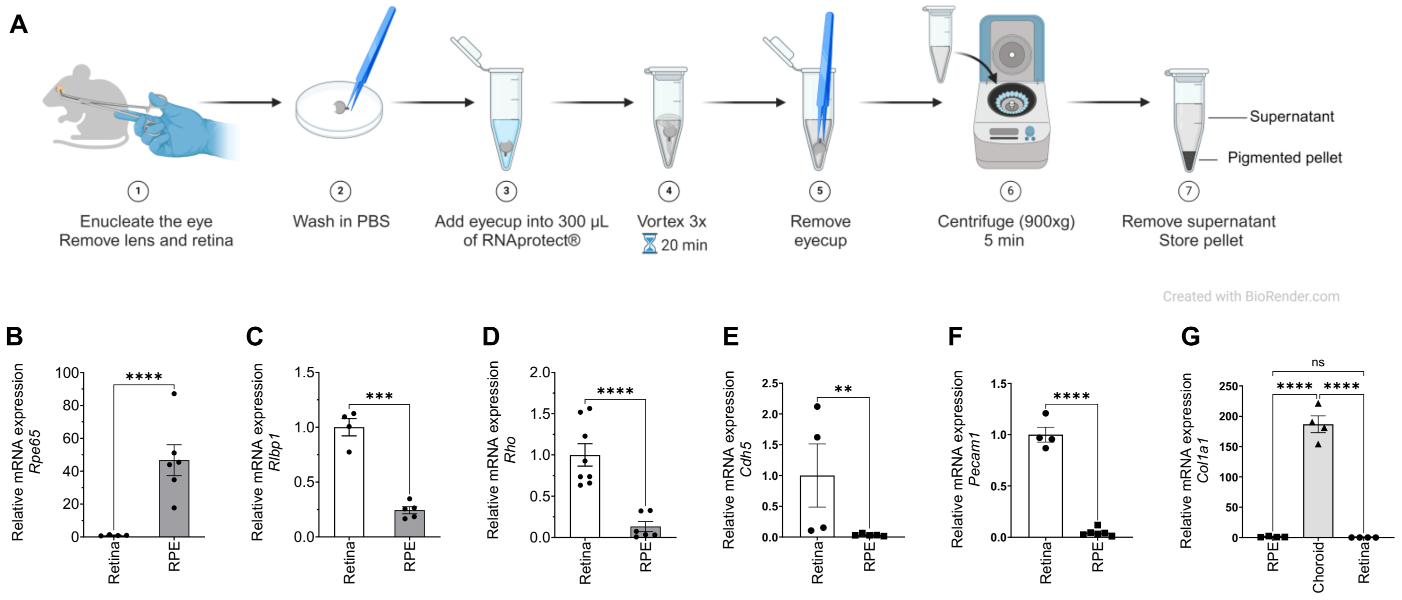

Supplement Figure S1: Validation of RPE cell RNA isolation by SRIRS method. (A) Schematic of the SRIRS RPE RNA isolation method [1] with the following steps: 1) Enucleate the eye, removing the lens and retina. 2) Wash the remaining posterior eyecup with PBS. 3) Immediately transfer the eyecup into a microcentrifuge tube containing 300 µL of RNAprotect® and incubate for 20 minutes at room temperature. 4) Briefly vortex twice during the 20 minutes period (e.g. at 7 minutes and 14 minutes), and once after the incubation time has passed to ensure most of the RPE cells are lysed. 5) Remove the eyecup using clean forceps. 6) Centrifuge for 5 minutes at 900 g. 7) Carefully aspirate the supernatant using a micropipette and store the pigmented pellet at -80°C for subsequent total RNA purification. (Figure created with BioRender.com.) **(B-F)** To evaluate RPE RNA enrichment and potential contamination with non-RPE-derived mRNAs in the SRIRS RNA from C57BL6/J mice, relative mRNA contents in retina and SRIRS RPE RNA were compared for **(B)** *Rpe65* (RPE marker gene, confirming RPE RNA enrichment), **(C)** *Rlbp1* (highly expressed by retinal Müller cells and by RPE), **(D)** *Rho* (a highly expressed rod photoreceptor-specific gene, confirming minimal retinal RNA contamination), and **(E)** *Cdh5* (highly expressed by retinal and choroidal endothelial cells) and **(F)** *Pecam1* (aka CD31, an endothelial cell marker), all normalized to 18S ribosomal RNA. **(G)** Relative *Col1a1* mRNA (highly expressed by choroidal stromal cells and choroidal smooth muscle cells [2]) contents in SRIRS RPE RNA, RNA from choroid/sclera, and retinal RNA, confirming lack of contamination by choroidal RNA in the SRIRS RPE RNA preparation. Bar graphs represent mean ± SEM. Statistical analysis was performed using one-way ANOVA with repeated measures followed by Šídák's multiple comparisons test. $**p < 0.01$; $***p < 0.001$; $****p < 0.0001$.

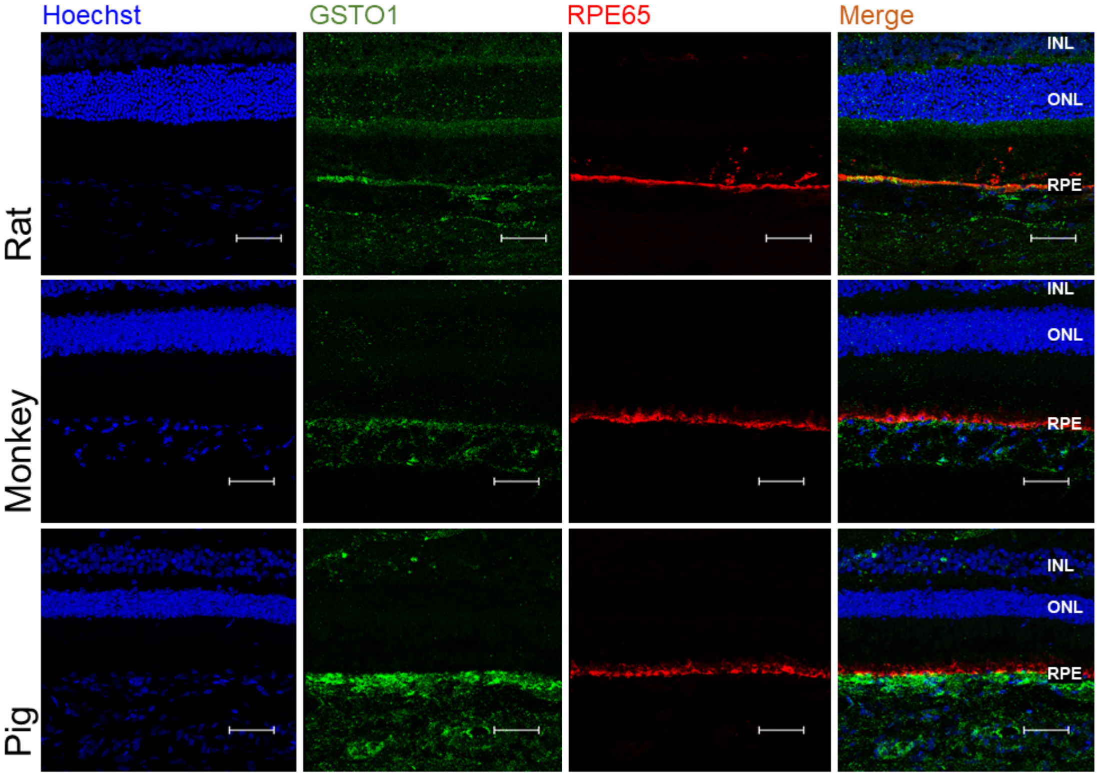

Supplemental Figure S2: GSTO1 immunofluorescence in the RPE of rat, monkey and pig. Immunofluorescence analysis of retinal sections of naïve Brown Norway rat, Rhesus monkey, and pig. Staining was performed to visualize cell nuclei (blue), GSTO1 IF (green), and IF of the RPE cell marker RPE65 (red). The right column of images shows merged overlays of the blue, green and red channels. Images were acquired using the Leica STELLARIS 8 FALCON Confocal Microscope, 40x objective. Scale bar = 50 µm.

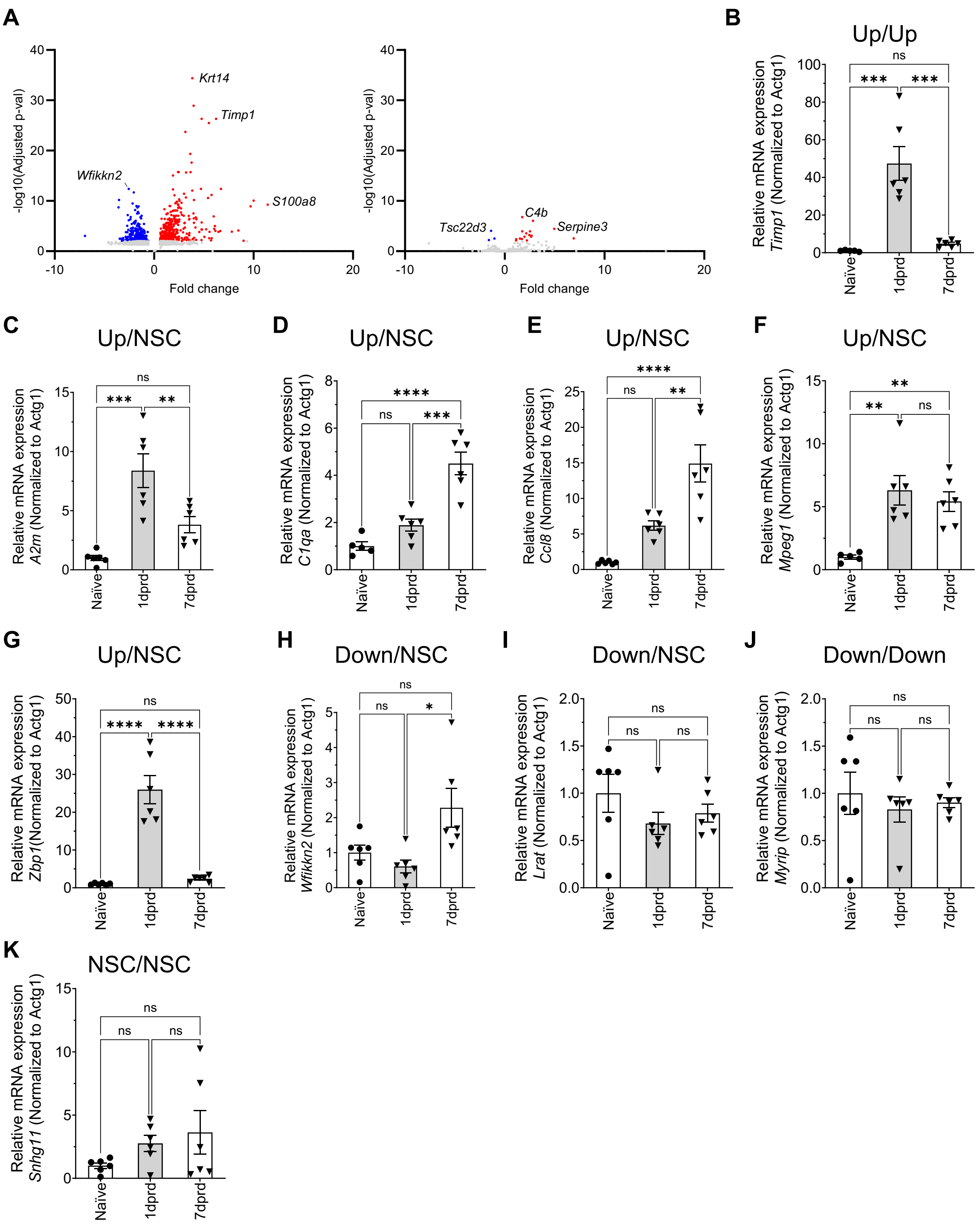

Supplement Figure S3: Validation of additional temporal gene expression changes after retinal detachment. **(A)** Volcano plots showing upregulated DEG (red), down-regulated DEG (blue) and non-significantly changed genes (NSC, gray) at 1 dprd (left) and 7 dprd (right). RNA samples were obtained from SRIRS RPE RNA preparations from naïve (n=5) RPE of C57BL6/J mice, as well detached RPE at 1 dprd (n=6) and 7 dprd (n=6). **(B-K)** QRT-PCR validation of DEG in temporal groups using *Actg1* as an internal control. Relative mRNA expression for: **(B)** *Timp1,* identified as a DEG upregulated at both 1 dprd and 7 dprd (Up/Up); **(C)** *A2m,* **(D)** *C1qa,* **(E)** *Ccl8,* **(F)** *Mpeg1,* and **(G)** *Zbp1,* identified as DEG up-regulated at 1 dprd and not significantly changed at 7 dprd (Up/NSC); **(H)** *Wfikkn2* and **(I)** *Lrat*, identified as DEG down-regulated at 1 dprd and not significantly changed at 7 dprd (Down/NSC); **(J)** *Myrip,* identified as down-regulated at both 1 and 7 dprd (down/down); and **(K)**

Snhg11, which was not significantly changed at either time post RD (NSC/NSC) in the RNAseq analysis. Bar graphs represent mean ± SEM. Statistical analysis was performed using one-way ANOVA with repeated measures followed by Tukey's post hoc test. **p < 0.01; ***p < 0.001; ****p < 0.0001. Results for *Myrip* and *Snhg11* mRNAs were confirmed using second sets of TaqMan assays for each (data not shown).

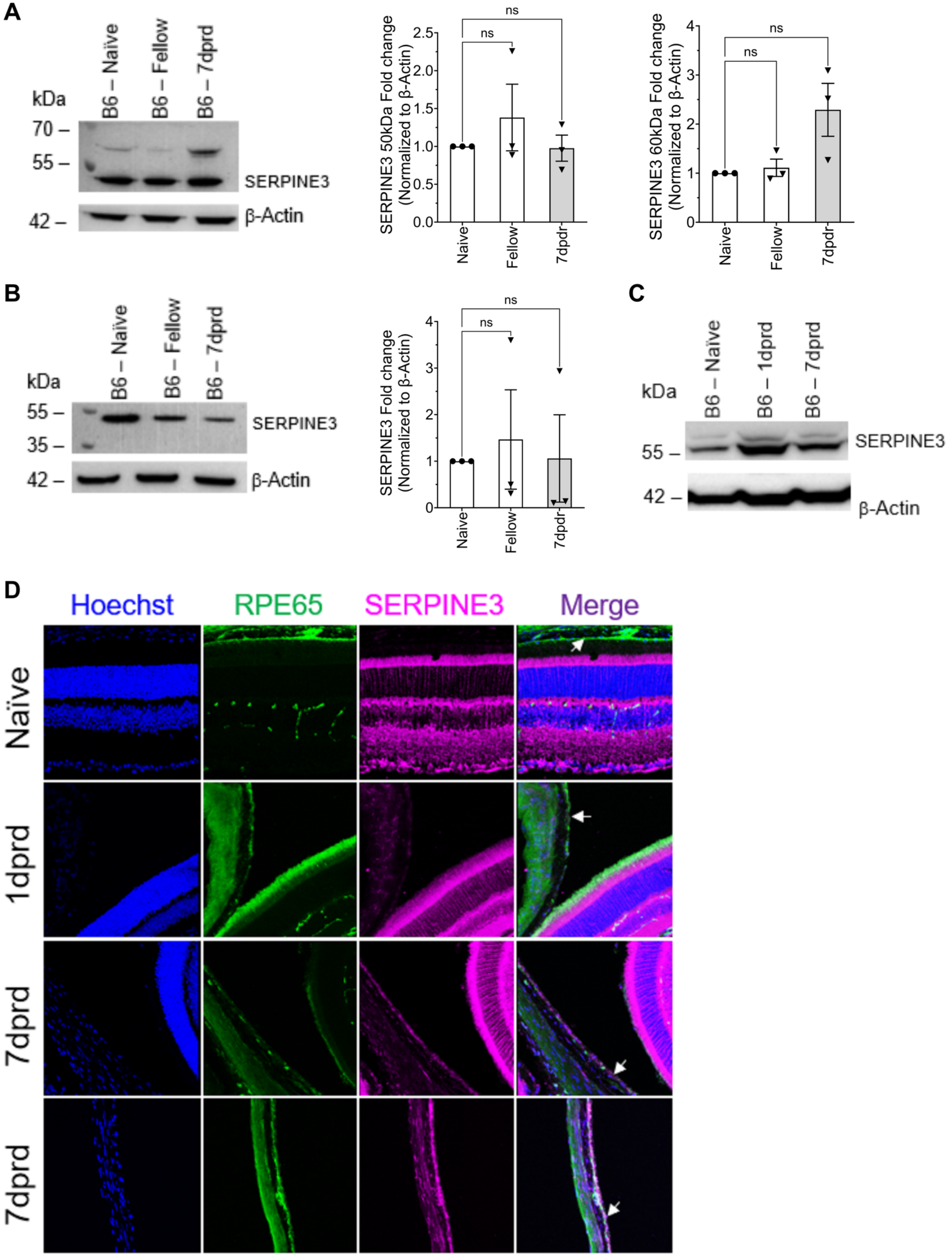
A
kDa
B6 – Naïve
B6 – Fellow
B6 – 7dprd
70 –
55 –
42 –
SERPINE3
β-Actin
SERPINE3 50kDa Fold change (Normalized to β-Actin)
SERPINE3 60kDa Fold change (Normalized to β-Actin)
ns
Naive
Fellow
7dpdr
B
35 –
SERPINE3 Fold change (Normalized to β-Actin)
C
B6 – 1dprd
D
Hoechst
RPE65
SERPINE3
Merge
Naïve
1dprd
7dprd

Supplement Figure S4: Results of examination of SERPINE3 protein expression changes after retinal detachment. Representative immunoblots of SERPINE3 in soluble protein preparations from eyecups of C57BL6/J mice under naïve, contralateral (fellow at 7 dprd), and 1 day detached (1 dprd) or 7 day detached (7 dprd) conditions. Blots are representative of 3 independent animals in each condition. **(A)** Represents blots and respective quantifications obtained with a rabbit polyclonal antibody (Atlas Antibodies Cat# HPA055804) raised against human SERPINE3 peptide (sequence: YVSEAIHKAKIEVLEEGTKASGATALLLLKRSRIPIFKADRPFIYFLREPNTGITVFFDRIQIIYQCLSSNKGSFVHYP, 79% homologous to mouse SERPINE3) and protein samples separated under non-reducing conditions, which resulted in two major bands (~50 kDa and ~60kDa) being detected. **(B)** Represents blots obtained with the Atlas anti-SERPINE3 peptide polyclonal antibody against protein samples separated under reducing conditions, which resulted in a single band (~52 kDa) being detected. **(C)** Represents blots obtained with a rabbit polyclonal SERPINE3 antibody (BOSTER Biological Technology, Cat#A18879, raised against human SERPINE3 recombinant protein fragment K90-F424, 66% homologues to mouse SERPINE3) with protein samples separated under reducing conditions. Note that the BOSTER company claims that the antibody reacts with human, rat and mouse SERPINE3 in western blotting and ELISA applications. In summary, we were unable to consistently demonstrate an increase in SERPINE3 protein in the 7 dprd RPE samples by western blotting using two different antibodies. **(D)** Immunofluorescence analysis (using the BOSTER SERPINE3 antibody) of retinal sections from C57BL6/J mice under naïve and detached (1 dprd and 7 dprd) conditions indicate an increase in anti-SERPINE3 IF colocalizing with the RPE65 protein after RD (arrow). Staining of cell nuclei (blue), and anti-RPE65 IF (green), and anti-SERPINE3 IF (magenta) shown. The right column of images represents merged overlay of the green, magenta, and blue channels. Note that no SERPINE3 IF was detected in RPE in sections from naïve samples. The bottom row of images focuses on the RPE layer to demonstrate co-localization of antiSERPINE3 with anti-RPE65. Also note that nonspecific binding of the antibody is suspected because of the strong IF signal in the retina, even though RNAseq comparison of naïve retina and RPE indicated that the RPE expresses 61-fold more Serpine3 mRNA than retina (data not shown). Also note that IF analysis with the Atlas SERPINE3 antibody did not result in IF signal detected in the RPE (data not shown).

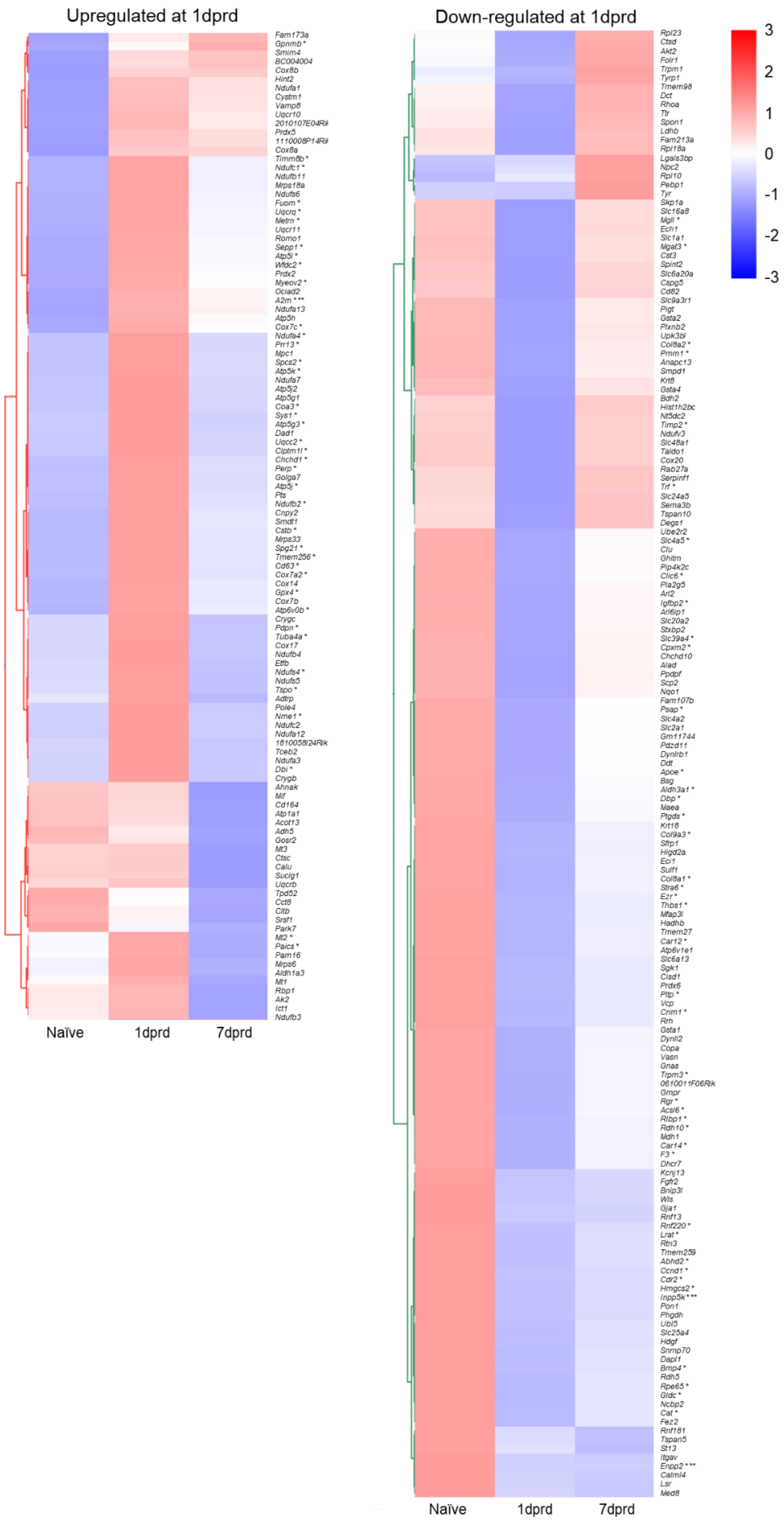
Upregulated at 1dprd
Naïve
1dprd
7dprd
Down-regulated at 1dprd
Naïve
1dprd
7dprd
3
2
1
0
-1
-2
-3

Supplement Figure 5: Effects of Retinal Detachment on RPE Signature Genes. Heatmap of Z-scores for TPM effect of retinal detachment of the 277 genes in the present mouse RPE transcriptome identified in this study that are in common with one or more previously published RPE signature gene lists (Figure 1B). *p < 0.05; **p < 0.01; ***p < 0.001, ****p < 0.0001.

Supplemental References:

1. Xin-Zhao Wang C, Zhang K, Aredo B, Lu H, Ufret-Vincenty RL: **Novel method for the rapid isolation of RPE cells specifically for RNA extraction and analysis.** *Exp Eye Res* 2012, **102:**1-9.
2. Lehmann GL, Hanke-Gogokhia C, Hu Y, Bareja R, Salfati Z, Ginsberg M, Nolan DJ, Mendez-Huergo SP, Dalotto-Moreno T, Wojcinski A, et al: **Single-cell profiling reveals an endothelium-mediated immunomodulatory pathway in the eye choroid.** *J Exp Med* 2020, **217**.
