# Supplementary figures and images for: The mouse retinal pigment epithelium mounts an innate immune defense response following retinal detachment
Source: J Neuroinflammation. 2024 Mar 25;21:74. doi: 10.1186/s12974-024-03062-2 (PMC10964713; doi:10.1186/s12974-024-03062-2)

Figure 3B

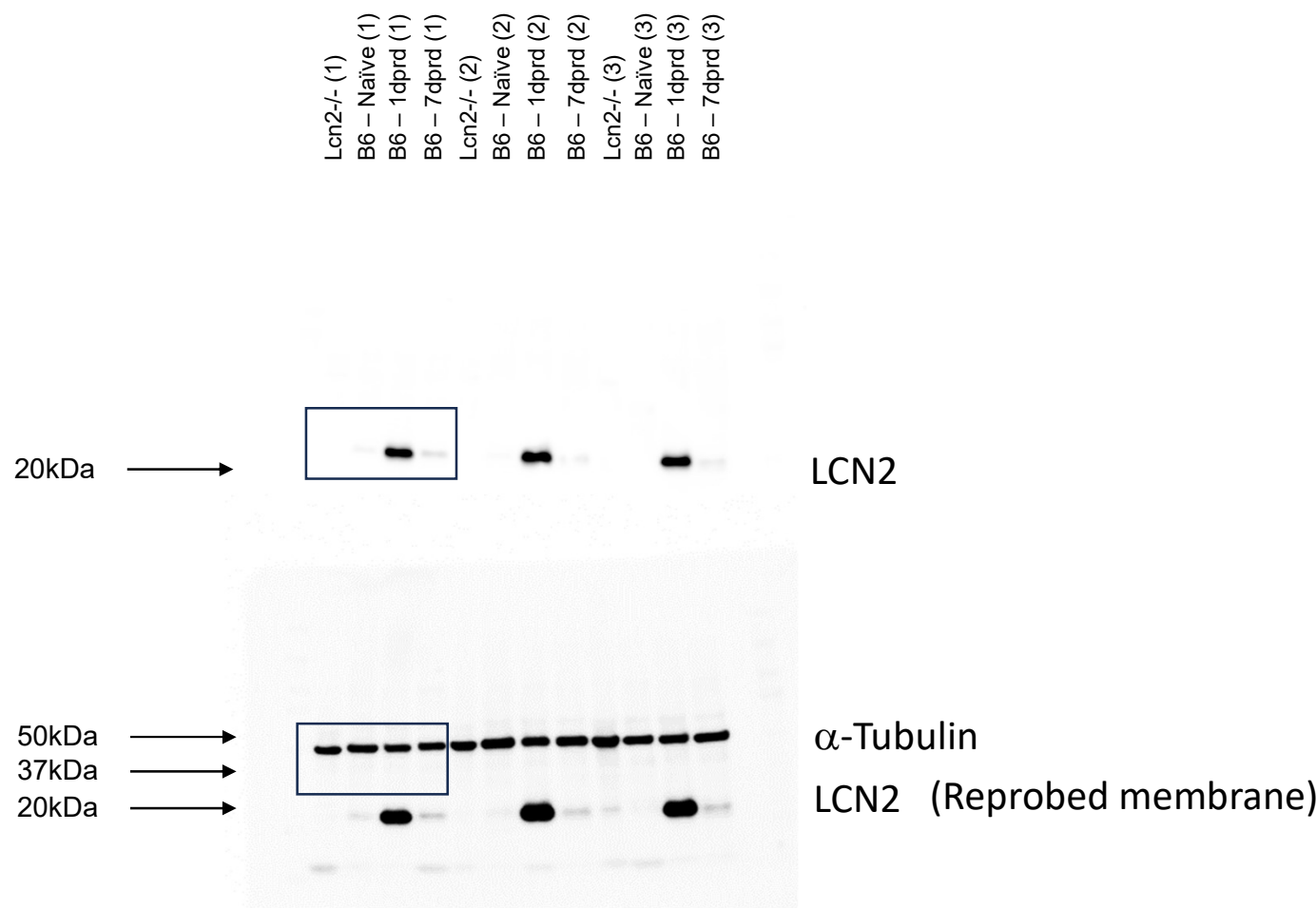

Figure 4B

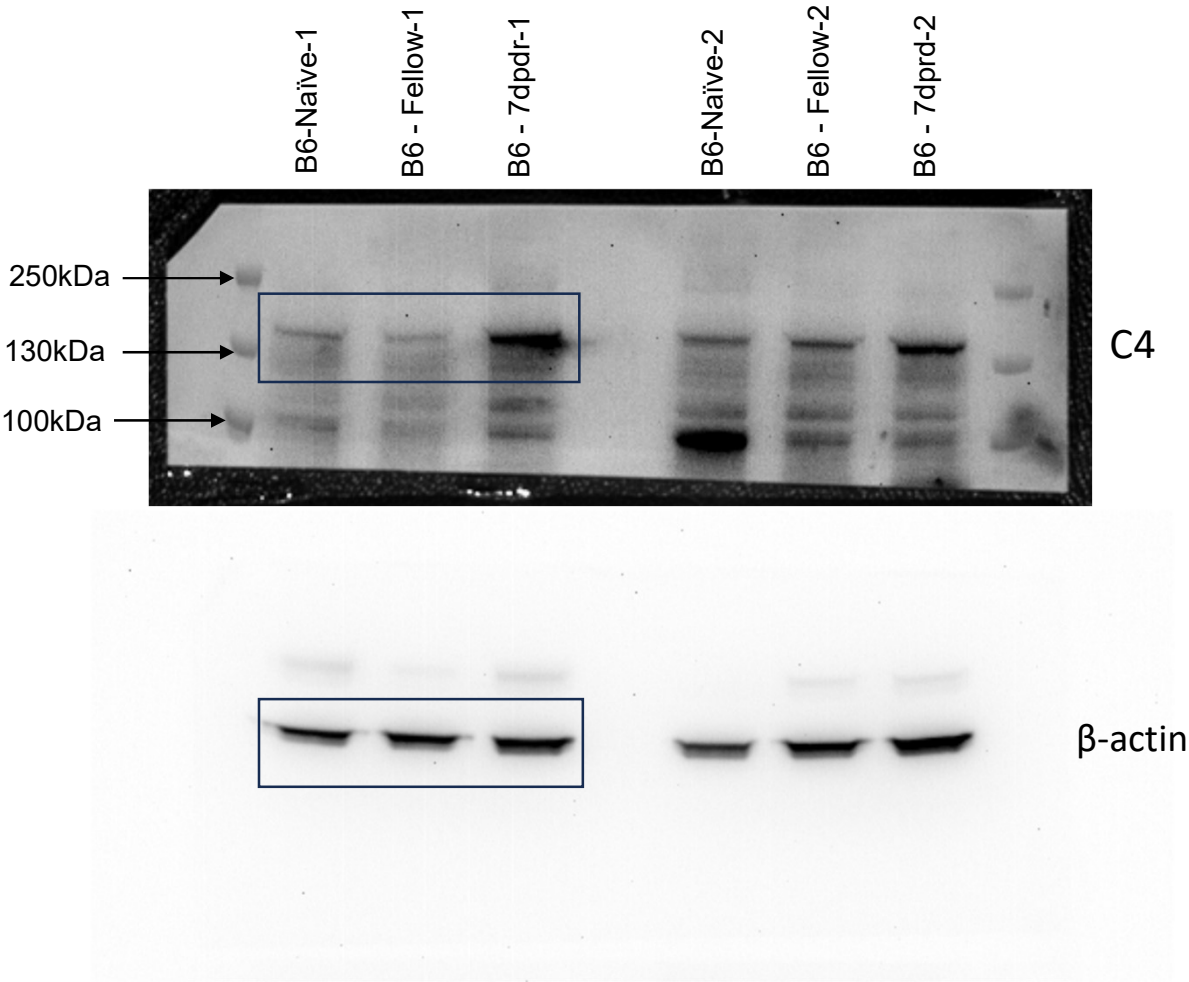

Supplement Figure S4A

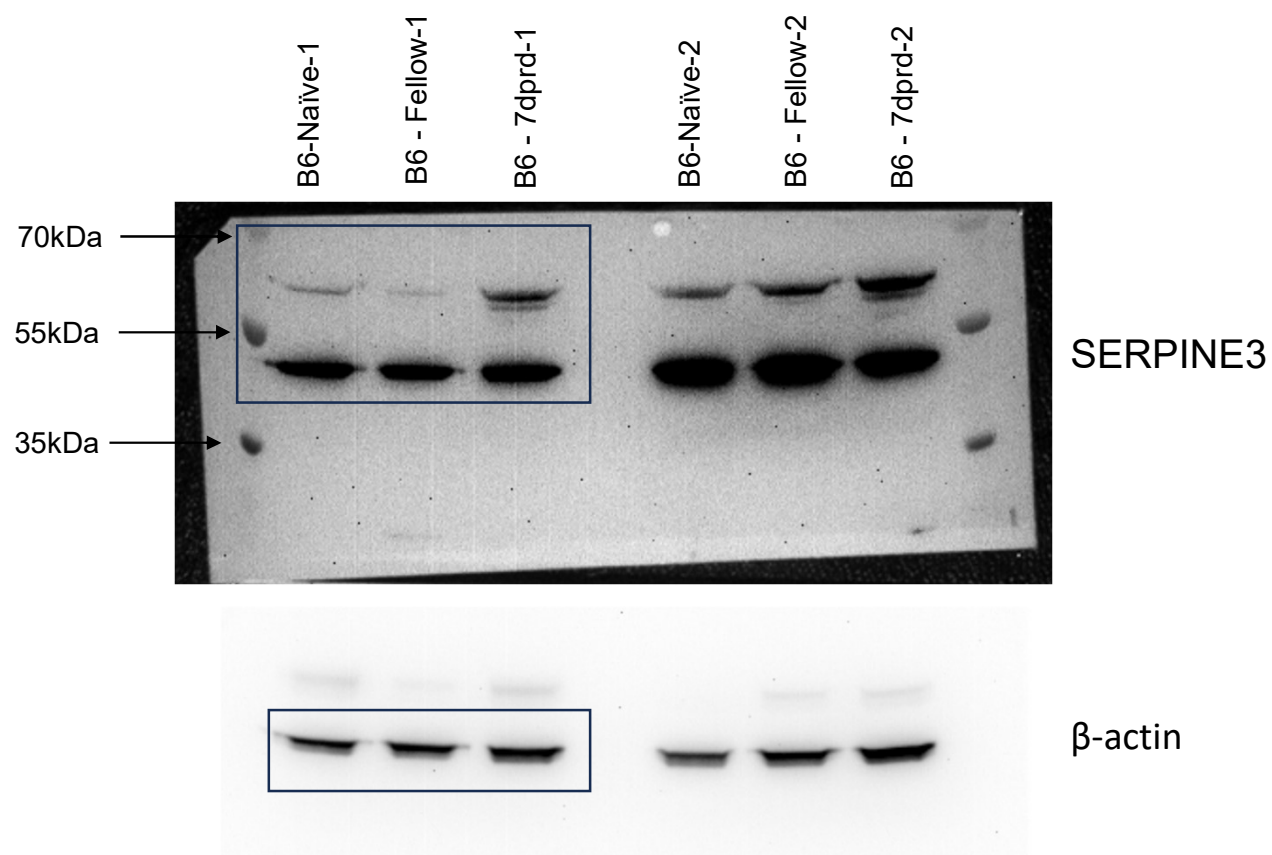

Supplement Figure S4B

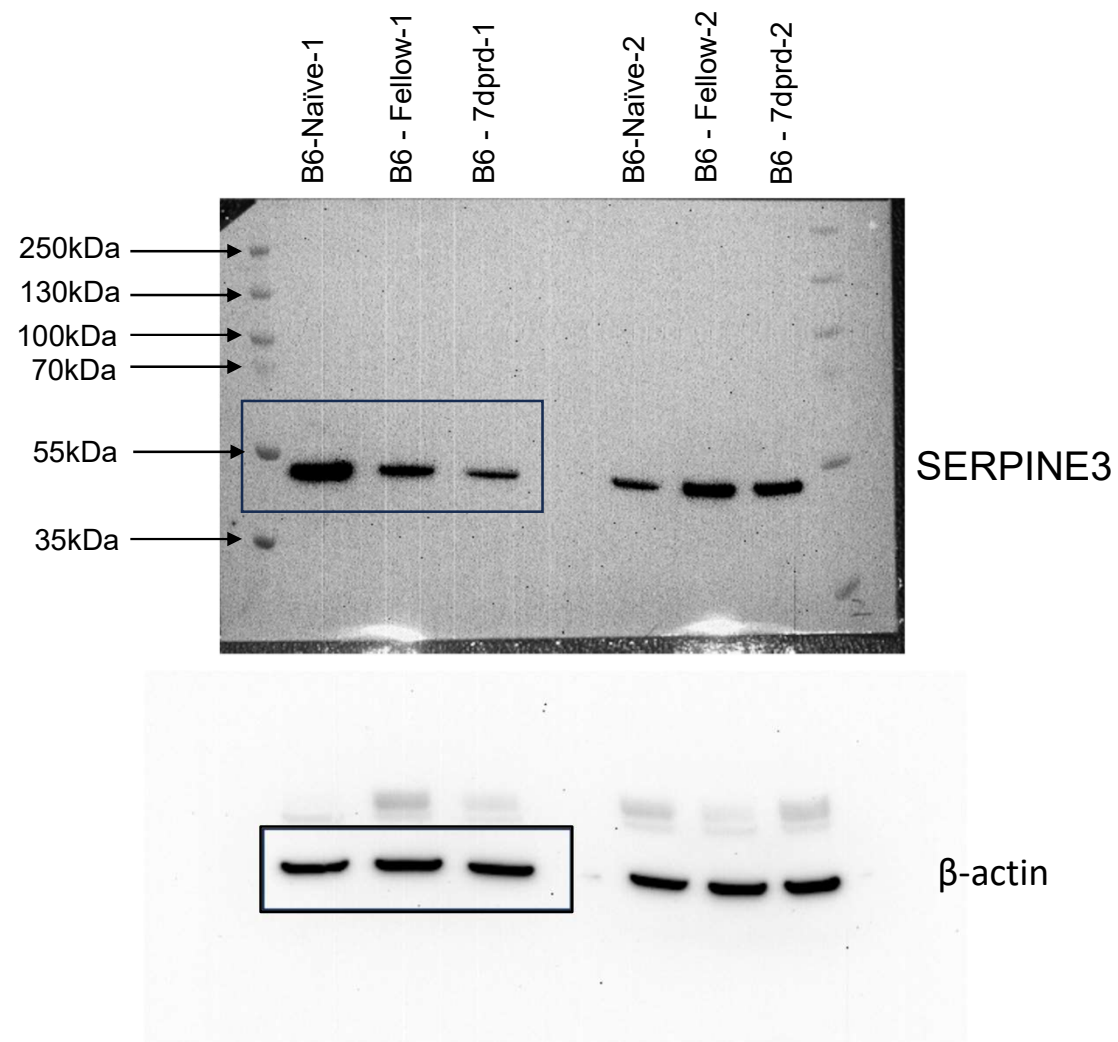

Supplement Figure S4C

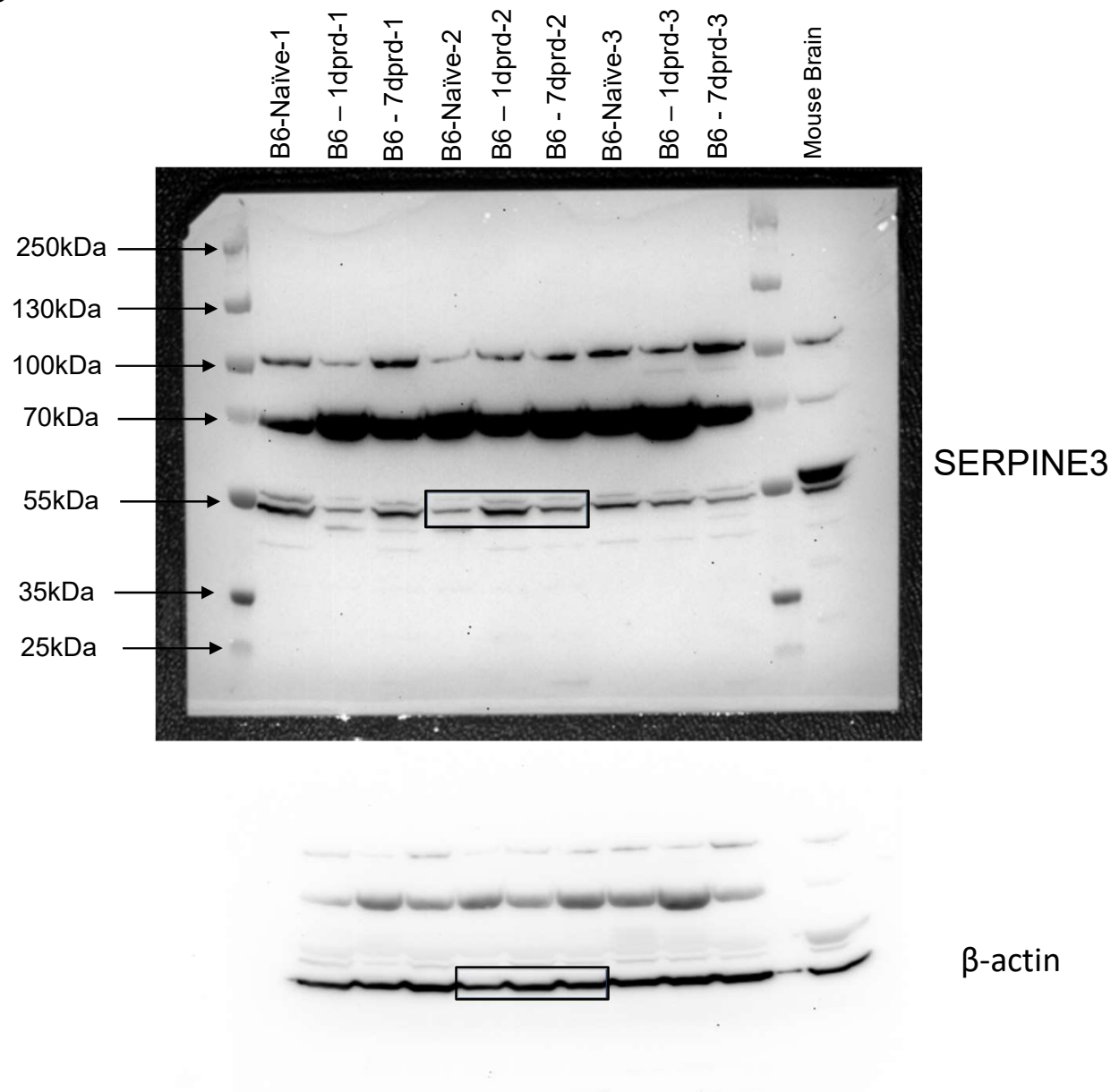

Supplement: Supplementary file 4 — Supplementary Material 4 [file 12974_2024_3062_MOESM4_ESM.pdf]
